# Study on biotransformation and absorption of genistin based on fecal microbiota and Caco-2 cell

**DOI:** 10.3389/fphar.2024.1437020

**Published:** 2024-10-09

**Authors:** Zhe Li, Yuqing Wang, Zicheng Wang, Dongxue Wu, Yuhao Zhao, Xun Gong, Quan Jiang, Congmin Xia

**Affiliations:** ^1^ Guang’an Men Hospital, China Academy of Chinese Medical Sciences, Beijing, China; ^2^ School of Pharmacy, Shandong University of Traditional Chinese Medicine, Jinan, China; ^3^ School of Pharmacy, Binzhou Medical University, Yantai, China

**Keywords:** genistin, probiotics, metabolites, 16S RNA sequencing, Caco-2 cell

## Abstract

**Introduction:**

Genistin, as a kind of natural isoflavone glycoside, has good biological activity, and its weak absorption makes it closely related to intestinal flora. However, the role of the intestinal flora is still unclear and whether the metabolites produced by the intestinal flora are absorbed systemically is also variable.

**Methods:**

Genistin was fermented for 24 h based on fecal bacteria fermentation technology. The components were qualitatively and quantitatively analyzed by HPLC and UHPLC-Q-Exactive Orbitrap Mass spectrometry. The composition of intestinal flora in fermentation samples from fecal bacteria was detected by 16S rRNA sequencing. Five representative probiotics were cultured *in vitro* and fermented with genistin to determine similarities and differences in genistin metabolites by different bacteria at different times. Finally, the absorption results of metabolites by fermentation were verified by a Caco-2 cell monolayer.

**Results:**

The HPLC results of fecal fermentation showed that genistein levels increased from 0.0139 ± 0.0057 mg/mL to 0.0426 ± 0.0251 mg/mL and two new metabolites were produced. A total of 46 metabolites following fecal fermentation were identified, resulting from various biotransformation reaction products, such as decarbonylation, hydroxylation, and methylation. Simultaneously, the 16S rRNA results showed that the intestinal flora changed significantly before and after fermentation and that the intestinal microorganisms in the control (Con) group and the fermentation (Fer) group showed a significant separation trend. Five genera, *Lactobacillus*, *Bifidobacterium*, *Parabacteroides*, *Sutterella*, and *Dorea*, were considered the dominant flora for genistin fermentation. The qualitative results of fermentation of genistin by five probiotics at different times showed that there were significant differences in small molecule metabolites by fermentation of different bacteria. Meanwhile, most metabolites could be identified following fecal bacteria fermentation, which verified the importance of the dominant bacteria in the feces for the biotransformation of components. Finally, the absorption results of the metabolites based on the Caco-2 cell monolayer showed that 14 metabolites could be absorbed into the circulation in vivo through the mesentery.

**Discussion:**

The small molecule metabolites of genistin by fermentation of fecal bacteria can be well absorbed systemically by the body. These studies provide a reference value for explaining the transformation and absorption of flavonoid glycosides in the intestine.

## 1 Introduction

In recent years, isoflavones have received extensive attention due to their nutritional and potential health benefits. As non-steroidal phytoestrogens and antioxidant polyphenol molecules, they have good activities in preventing hormone-dependent diseases (Rekha and Vijayalakshmi, 2010). Genistin (4′,5,7-trihydroxyiso-flavone 7-glucoside), a natural isoflavone, is widely distributed in Leguminosae (Barnes et al., 2011; Ganai and Farooqi, 2015; Islam et al., 2020). It exerts many activities including anti-inflammatory, antioxidant, anti-viral, antiatherosclerosis, and antihyperlidemic effects (Sebastián Domingo and Sánchez Sánchez, 2018; Rasheed et al., 2022; Wang et al., 2023). Simultaneously, compared with other isoflavone glycosides, genistin has the advantages of rich source, stable nature, good activity and small toxic and side effects (Liang et al., 2018). However, isoflavonoid glycosides cannot be absorbed into the human body through the gastrointestinal barrier (Wang H. et al., 2022). Studies have reported that isoflavone glycosides can be hydrolyzed by enzymes in the body into numerous metabolites that are absorbed into circulation, and these enzymes come from the intestinal flora (Xin et al., 2019). Furthermore, it is worth noting that the interaction between genistin and intestinal flora *in vivo* has been rarely reported in previous literature studies.

The intestinal flora is the largest microecosystem in the human body and has a significant impact on body material and energy metabolism (Yang and Tsai, 2019; Yao et al., 2019). The functional aspects of the normal human gut microbiota comprise metabolic and nutritional functions, antimicrobial protection, maintenance of intestinal mucosa integrity, and regulation of the immune response (Ashwood et al., 2018; Feng et al., 2020). It is worth noting that an increasing number of studies have focused on the role of the intestinal flora in glycoside metabolism. Lactase-phenylpropanol hydrolase (LPH) produced by the intestinal flora hydrolyzes quercetin 3-o-glucoside (Q3G) and quercetin 4′-o-glucoside (Q4′G), as well as genistein and daidzein monoglucosides *in vitro* to produce the corresponding aglycones, and cytoplasmic β-glucosidase (CBG) can hydrolyze Q4′G, genistin, and daidzin (Ge et al., 2022). Interestingly, the intestinal flora in the feces of humans or animals, contain abundant enzymes and exert a strong enzymatic hydrolysis activity for carbohydrates contained in plants (Jandhyala et al., 2015; Hu et al., 2022). However, as the main metabolic organ, the intestinal tract has not been studied extensively or examined systematically in previous studies, resulting in incomplete results. Recently, Ultra-High-Performance Liquid Chromatography Quadrupole Exactive Orbitrap Mass Spectrometry (UHPLC-Q-Exactive Orbitrap MS) has been used in the discovery of trace substances *in vivo* based on its high resolution and high throughput (Li et al., 2022; Liu et al., 2022). In addition, a scientific analysis strategy is also a critical factor restricting the identification of metabolites. Potential screening methods including high-resolution extracted ion chromatography (HREIC), multiple mass defect filtering (MMDF), neutral loss filtering (NLF), and diagnostic product ions (DPIs) can be applied for the detection and identification of metabolites (Liu et al., 2017; Murota et al., 2018).

Genistin can be hydrolyzed into other metabolites by the intestinal flora in the body. However, it is unclear which bacteria are involved and whether the metabolites produced can be absorbed by the human body. Therefore, in this study, the components of fecal bacteria fermentation were qualitatively and quantitatively measured using UHPLC-Q-Exactive Orbitrap MS and HPLC technology. Correspondingly, the changes in intestinal flora after fecal bacteria fermentation were analyzed by 16S rRNA sequencing technology. Subsequently, five representative probiotics were cultured *in vitro* and fermented with genistin to determine the similarities and differences of the genistin metabolites by different bacteria at different times. Finally, the absorption of fermented genistin metabolites was verified using the Caco-2 cell monolayer. These results have not been reported in the previous literature. This study revealed similarities and differences in the transformation of genistin by the gut microbiota in the feces.

## 2 Materials and methods

### 2.1 Chemicals and reagents

Methanol, acetonitrile, and formic acid of HPLC grade were purchased from Kemiou (Tianjin, China). General anaerobic medium (GAM) broth, DeMan Rogosa Sharpe (MRS) Liquid medium, vitamin K1, and hematin chloride were purchased from Hopebio (Qingdao, China). The standard materials of Genistin, genistein, daidzein, and daidzein were purchased from Chengdu Must Biotechnology Co., LTD (Shenzhen, China). All standard products were tested by high-performance liquid chromatography with purity greater than 98%. The pure water for this experiment was obtained from Wahaha (Hangzhou, China). *Lactobacillus*, *Bifidobacterium adolescentis*, *Bifidobacterium longum*, *Lactobacillus plantarum F1,* and *L. plantarum B2* were borrowed from the Pathogenic Biology Laboratory of Yantai Campus of Binzhou Medical College. The intestinal epithelial cell line Caco-2 (derived from human colorectal adenocarcinoma cells) was purchased from Wuhan Punosai Life Science and Technology Co., Ltd (Wuhan, China). Heat-inactivated foetal bovine serum (FBS) and Hank’s Balanced Salt Solution (HBSS) were obtained from Sigma Aldrich (St. Louis, MO, United States). 4′,6-diamidino-2-phenylindole (DAPI), and alkaline phosphatase kits (ALP) were purchased from Jiancheng Bioengineering Institute (Nanjing, China). Fluorescein Isothiocyanate (FITC) was purchased from NeV Biotech Co., LTD (Chongqing, China).

### 2.2 Fermentation of genistin by fecal bacteria

A total of 10 male Sprague-Dawley (SD) rats (SPF level, weighing 180–200 g) were purchased from Jinan Pengyue Experimental Animal Breeding Co., Ltd [Shandong, China, SYXK (RU)2019-0003]. 10 g fresh feces of normal rats were taken, and the fecal samples were mixed with normal saline at a ratio of 1:5 (w/v). The sterilized glass rod was stirred for 10 min to promote the bacterial solution to fully enter the normal saline. Subsequently, gauze was used to filter out fecal impurities without bacteria. Meanwhile, the bacterial liquid was retained for use. Correspondingly, 49 g GAM medium was dissolved in 1 000 mL distilled water and autoclaved at 121°C for 15 min. After cooling, 1 mg vitamin K1 and 5 mg hemin were added to the medium. Subsequently, the normal fecal suspension (30 mL) was mixed with GAM medium 1:1, and 40 mL was added to 20 mg of genistin standard to prepare a fermentation broth (0.5 mg/mL), and 4 mL of fermentation broth was added to each culture dish. The petri dish was put into an anaerobic culture bag and fermented at 37°C. Simultaneously, 100 rpm was used in the bacterial incubator to promote the full mixing of genistin and fecal bacteria. Samples were taken at 0 h and 24 h, respectively, and a control group (fecal suspension + GAM medium 1:1) was set up (Ramakrishna, 2013). Five parallel samples were prepared for each of the above, and each group of parallel samples was packed in the same anaerobic bag to obtain accurate results. The animal experiment was approved by the Ethics Committee of Guang’an Men Hospital, China Academy of Chinese Medical Sciences (Approval No. 2023-203-KY). The animal facilities and protocols complied with the Guide for the Care and Use of Laboratory Animals (United States National Research Council, 1996; Chinese Pharmacopoeia Commission, 2020).

1.0 mL of parallel samples was taken from each group, and was mixed with cold methanol at a ratio of 1:4. The solution was placed at 4°C overnight for protein precipitation and fermentation termination, and was centrifuged at 4,500 r/min for 10.0 min. The supernatant was diluted to 5.0 mL. Meanwhile, 1 mL of the supernatant was used for HPLC analysis. Next, 1.0 mL of supernatant was taken for UHPLC-Q-Exactive Orbitrap MS analysis.

### 2.3 Biotransformed sample analysis by HPLC

All biotransformed samples were quantified using an HPLC system, which consisted of two LC-20AT solvent delivery systems, a DGU-20A3R degasser, a CTO-20A column oven, a SIL-20AXR autosampler, an SPD-20A UV detection, and a Labsolution workstation (Shimadzu, Kyoto, Japan). Based on the method in the Chinese Pharmacopoeia Commission, 2020, a Kromasil 100-5-C18 column (250 mm × 4.6 mm, 5 μm, AKZO NOBEL, Bohus, Sweden) was used as the separating medium. The mobile phase consisted of acetonitrile (A) and 0.2% formic acid solution (B). The gradient elution program was set as follows: 0–20 min, 20%–40% A; 20–30 min, 40% A; and 30–40 min, 40%–20%A. The UV absorption spectra were measured using a flow rate of 1.0 mL/min, the column temperature of 25°C, the wavelength of 260 nm, and the injection volume was 10.0 μL.

### 2.4 Biotransformed sample analysis by UHPLC-Q-Exactive Orbitrap MS

Genistin and its metabolites were obtained and performed on a DIONEX Ultimate 3000 UHPLC system (Thermo Fisher Scientific, MA), which is equipped with a binary pump, an automated sampler, and a column compartment. The chromatographic separations were performed on a Waters ACQUITY BEH C18 column (2.1 mm × 100 mm, 17 μm, Waters, Milford, MA, United States). The mobile phase consisted of 0.1% formic acid aqueous solution (A) and acetonitrile (B) at a flow rate of 0.25 mL/min. The linear gradient procedure was described as follows: 0–2 min, 5%–20% B; 2–5 min, 20%–25% B; 5–7 min, 25%–30% B; 7-8 min, 30%–45% B; 8–9.5 min, 45%–55% B; 9.5–10.5 min, 55%–60% B; 10.5–12 min, 60%–70% B; 12-13 min, 70%–95% B; 13–15 min, 95%–5% B; 15–17 min, 5% B. The injection volume was 2.0 μL. The UHPLC-Q-Exactive Orbitrap MS and tandem mass spectrometry (MS/MS) spectra were obtained using a Q-Exactive Orbitrap mass spectrometer with electrospray ionization (ESI) (Thermo Fisher Scientific, MA). An analytical run was performed by mass spectrometry in positive and negative ion modes. The parameters were listed as follows: nitrogen (purity ≥99.99%) served as the sheath gas and auxiliary gas. The flow of sheath gas (nitrogen) was 35 arb and auxiliary gas (nitrogen) was 10 arb. The capillary temperature was 320°C, the probe heater temperature was 325°C, and the cone voltage for ESI was 3 800/3 500 V (±). The Orbitrap analyzers were obtained high-resolution mass spectra with a full scan in *m/z* 70–1050 mass range at a resolution of 70,000 in MS and a resolution of 17,500 in dd MS/MS. The collision energy was set to 45% of the normalized collision energy to generate adequate fragment ions.

### 2.5 16S rRNA gene-based amplicon sequencing and bioinformatic analysis

Microbial marker-gene sequence data can be used to generate comprehensive taxonomic profiles of the microorganisms present in a given community and for other community diversity analyses (Hall and Beiko, 2018). Consequently, total genomic DNA of intestinal flora was extracted from genistin-fermented feces for 16S rRNA sequencing. Illumina NovaSeq platform was used to sequence the library. Trimmomatic 0.33, Cutadapt 1.9.1, USEARCH 10.0, and UCHIME 4.2 were used to concatenate and filter the raw data, resulting in high-quality sequences for subsequent analysis. USEARCH 10.0 was used to cluster Reads, the similarity was 97.0%, and OTU was obtained (Tan et al., 2023). The observed Chao1 richness estimator, Shannon diversity index, and Faith phylogenetic distance were used to estimate the microbial diversity in the samples (Wang Z. et al., 2022). Then Silva (http://www.arb-silva.de) was used as the reference database, and the feature sequences were labeled with a naive Bayes classifier to obtain the species classification information. QIIME2 2020.6 was used for alpha and beta diversity analysis. Cluster analysis heat map is an intuitive visualization method for analyzing the distribution of experimental data, which can be attributed to controlling the quality of experimental data and visualizing the different data (Santiago and Olesen, 2021). Therefore, 20 kinds of bacteria were analyzed by cluster analysis heat map.

### 2.6 Fermentation of genistin by five probiotics *in vitro*

Five probiotics were added to MRS Liquid medium and incubated for 24 h in the constant temperature shaker incubator at 37°C and 180 r/min. Following two consecutive generations of activation, the strains were inoculated into MRS Liquid medium at 10% volume (vol) and cultured at 37°C for 24 h, the probiotic mother liquor was prepared for use. Subsequently, five kinds of probiotic mother liquor (10% vol, 3 mL) were taken, 3.0 mg genistin was added. Furthermore, MRS Liquid medium was added and the total volume reached 30.0 mL, the fermentation was carried out at 37°C. Subsequently, the fermentation solution was collected at different time points (0, 1, 2, 3, 4, 6, 8, and 10 days). Meanwhile, all the fermentation samples were inactivated by high temperature after removal. Then, the dried samples were redissolved with 10 mL methanol and transferred to EP tubes for centrifugation (5 000 rpm, 10 min). The supernatant was transferred to a 10 mL volumetric flask and diluted with methanol. Finally, after all the samples were obtained, 1 mL solution was taken for HPLC and UHPLC-Q-Exactive Orbitrap MS analysis, respectively. The analysis method used was consistent with the fermentation of genistin feces. Meanwhile, the precision, repeatability, and stability of the results were investigated.

### 2.7 Absorption validation analysis based on the Caco-2 cell monolayer

Caco-2 cells of 10–20 passages were selected and digested to prepare a cell suspension with a density of 2 × 10^5^ cells/mL. The cell suspension was inoculated into a 12-well Transwell insert culture chamber with polycarbonate membrane material. 0.5 mL cell suspension was immediately added to the top side, and the inoculation amount was 1 × 10^5^ cell/insert. Correspondingly, 1.5 mL of complete medium was added to the basal side, and the apical (AP) side and basolateral (BL) side medium were replaced on the second day after inoculation. Subsequently, the AP and BL medium were changed every other day within a week, and the AP and BL medium were changed every day after a week until the cells formed a monolayer model (Riedel et al., 2006). After about 21 days of culture, Caco-2 cells in Transwell chamber were automatically differentiated into a single cell membrane. Meanwhile, the cell resistance, alkaline phosphatase activity, and laser confocal observation were continuously measured to verify whether the monolayer model was dense (Ding et al., 2014, Hu et al., 2021). The absorption experiment of genistin fermentation broth was carried out when the transmembrane resistance of Caco-2 cell monolayer tended to be stable. The complete medium on the AP and BL was sucked away, and then the cell monolayer was washed twice with Hank’s buffer. 0.5 mL and 1.5 mL of Hank’s buffer were added to the top side and the base side, respectively. After incubation in 5% CO_2_ incubator at 37°C for 30 min, the buffer was sucked away with a pipette. 0.5 mL fermentation broth was added to the AP, and 1.5 mL Hank’s buffer was added to the BL. The culture was placed in an incubator at 5% CO_2_ and 37°C. The liquid on the AP and the BL was taken out at 120 min. The liquid on both sides was evaporated and dissolved in methanol for UHPLC-Q-Exactive Orbitrap MS determination. All experiments were performed three times in parallel to obtain accurate results.

The cytotoxicity of Caco-2 cells was determined by 3-(4,5)-dimethylthiahiazo (-z-y1)-3,5-di- phenytetrazoliumromide (MTT) assay. Caco-2 cells in logarithmic phase were collected and the concentration of cell suspension was adjusted to 1 × 10^5^ cells/mL. Meanwhile, 100 μL of each well was inoculated into 96-well plates and incubated in 5% CO_2_ at 37°C for 24 h in an incubator. Furthermore, 100 μL of 1–10 mM fermentation broth containing 0.3% dimethyl sulfoxide (DMSO) was added and incubated for another 24 h. In addition, 20 μL of 5 mg/mL MTT and 5% CO_2_ were added to each well, and the supernatant was discarded after incubation at 37°C for 4 h. 150 μL DMSO was added to each well and the crystals were fully dissolved by shaking on a shaker at low speed for 10 min. Subsequently, the OD value at the wavelength of 595 nm was measured with a microplate reader. The zero setting well and the control well were set, and all experiments were performed in parallel three times to obtain accurate results.

### 2.8 Statistical analysis

The statistical analysis of the data was performed using SPSS 22.0 software (Chicago, IL, United States). An unpaired Student’s t-test was performed for a two-group comparison. For multiple comparisons, ANOVA was used. *p* < 0.05 was defined as statistically significant. The statistical analyses and figures were performed using GraphPad Prism 8.0 software (Santiago, MN, United States). The Thermo Xcalibur 2.1 workstation was used for data acquisition and processing. The parameters were set as follows: C [0–60], H [0–120], O [0–30], S [0–5], N [0–5], and the ring double bond (RDB) equivalent value [0–20]. Accurate mass measurements were set within a mass error of ±5 ppm.

## 3 Results

### 3.1 HPLC study of genistin conversion by fecal bacteria *in vitro*

**FIGURE 1:**
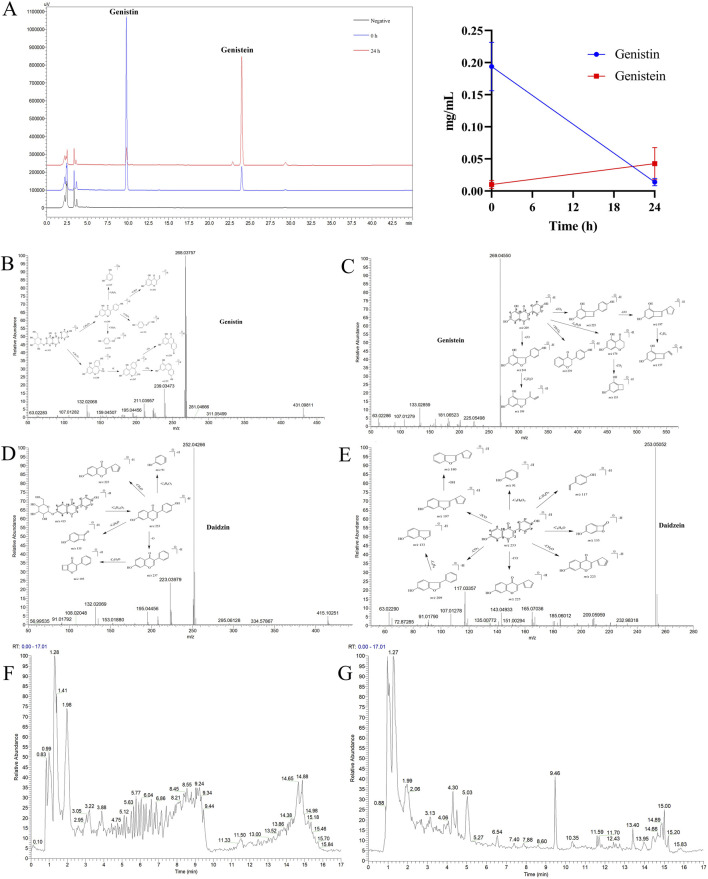
**(A)** HPLC analysis of the biotransformation product of genistin (left: the chromatogram of biotransformation products; right: the line chart showing the trend of biotransformation products; n = 5, data are presented as mean ± SD). Metabolic profiles of four templates: **(B)** Genistin. **(C)** Genistein. **(D)** Daidzin. **(E)** Daidzein. **(F)** Total ion current diagram of genistin fecal fermentation in positive ion mode. **(G)** Total ion current diagram of genistin fecal fermentation in negative ion mode.

The HPLC method for genistin was based on the Chinese Pharmacopoeia Commission (2020), and the methodological results are shown in Supplementary Table S1. The *in vitro* biotransformation products of genistin were analyzed by HPLC, and the results are shown in Figure 1A. The retention times of genistin and genistein were 9.8 min and 24.1 min, respectively. These levels were inconsistent with transformation. Genistin decreased from 0.1938 ± 0.0378 mg/mL at 0 h to 0.0102 ± 0.0063 mg/mL at 24 h, suggesting that genistin was transformed into other metabolites. Meanwhile, genistein increased from 0.0139 ± 0.0057 mg/mL at 0 h to 0.0426 ± 0.0251 mg/mL at 24 h. These results indicated that genistin had been converted mainly into genistein. However, there was still some genistein present in the fermentation system. This phenomenon can be attributed to the incomplete fermentation time. Surprisingly, compared to the negative control, genistin was biotransformed at the beginning of fermentation, which verified the rapid occurrence of the reaction. Similarly, the level of genistin was significantly lower than that of genistein at 24 h, indicating that intestinal flora could efficiently transform genistin. Coincidentally, two new metabolites were produced within 24 h of fermentation. They were significantly eluted at 10.8 min and 26.5 min, respectively. However, the chemical information of the two metabolites was vague.

### 3.2 The establishment of analytical strategy

In this study, a comprehensive and effective strategy was established to discover and identify genistin metabolites by UHPLC-Q-Exactive Orbitrap MS. Firstly, a high-quality full scan was performed with a resolution of 70,000 FWHM. Secondly, high-resolution extracted-ion chromatography was applied to withdraw the candidate data from positive and negative ion modes. Then, the candidate ions were systematically mined based on the common biological reactions and the reported metabolites in the literature. Those screened ions that we considered useful were added to the parent ion list (PIL) to obtain more accurate MS^2^ information for structure identification. Finally, the exact structures of these metabolites were resolved based on the exact molecular weight, fragmentation mode, DPIs, and information in the literature.

The composition types and mass spectra information on genistin were collected from the literature and analysis results by the established strategy. Furthermore, the MMDF metabolic templates were of significance in identifying those metabolites present at low levels. In this study, four templates were set in parallel to encircle the metabolites: (1) Genistin (*m/z* 431) and its conjugation templates (*m/z* 461 for hydroxylation and methylation); (2) Genistein (*m/z* 269) and its conjugation templates (*m/z* 241 for decarbonylation, *m/z* 271 for carbonyl hydrogenation, *m/z* 401 for arabino glycosylation); (3) Daidzin (*m/z* 415) and its conjugation templates (*m/z* 411 for dehydrogenation, *m/z* 472 for glycine conjugated); (4) Daidzein (*m/z* 253) and its conjugation templates (*m/z* 283 for hydroxylation and methylation). In addition, some metabolites were also set as new templates when these metabolites were found during the subsequent identification or when the current templates did not cover the metabolic profiles of genistin. The mass spectrum information for four prototype drugs (genistin, genistein, daidzin, and daidzein) was collected and resolved via the established analysis strategy. The metabolic profiles of genistin, genistein, daidzin, and daidzein are shown in Figures 1B–E. The total ion current diagram of genistin fecal fermentation is shown in Figures 1F, G.

### 3.3 Identification of metabolites

**FIGURE 2:**
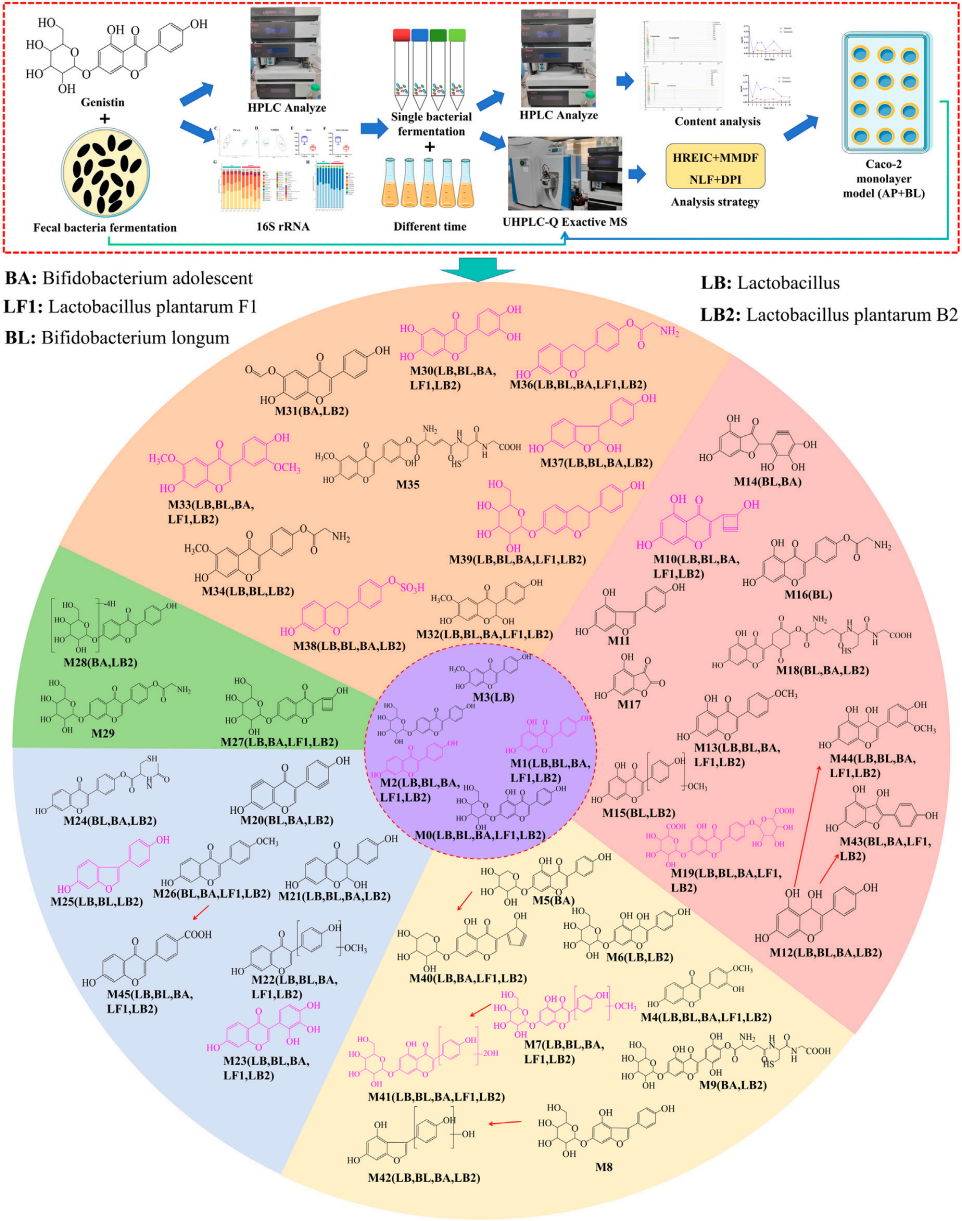
The biotransformation profile of genistin and the graphical summary of the entire analysis process. Purple: metabolite template. Different color regions in the circle represent the metabolites identified by each template (Orange: glycitein; Pink: genistein; Light yellow: genistin; Light blue: daidzein; Green: daidzin). Pink structural formula: Caco-2 cell monolayer absorption into the BL of the metabolites. LB, *Lactobacillus*; BL, Bifidobacterium longum; BA, Bifidobacterium adolescent; LF1, *Lactobacillus* plantarum F1; LB2, *Lactobacillus* plantarum B2.

A total of 46 metabolites were identified in the fermentation of fecal bacteria and genistin, involving a series of reactions such as decarbonylation, hydroxymethyl loss, carbonyl hydrogenation, and glycine binding (Table 1). Simultaneously, the biotransformation pathways of all metabolites are shown in Figure 2.

**TABLE 1:** LC-MS/MS data of genistin metabolites identified by fecal fermentation.

Peaks	Rt/min	Formula	Theoretical mass (m/z)	Experimental mass (m/z)	Error/ppm	Ion mode	MS^n^ fragment ions	Identification
M0	5.10	C_21_H_19_O_10_	431.09837	431.09799	−0.881	[M-H]^−^	268(100),269(63),267(18),239(15),431(7),211(7),132(7),240(7),223(4),212(4)	Genistin
M1	9.46	C_15_H_9_O_5_	269.04554	269.04553	−0.062	[M-H]^−^	269(100),270(19),133(16),159(8),63(8),107(6),183(5),201(3),135(3),180(3),157(3),196(3),169(2)	Genistein
M2	7.40	C_15_H_9_O_4_	253.05063	253.05054	−0.364	[M-H]^−^	253(100),254(20),224(8),209(6),91(5),133(5),208(5),225(4),135(4),197(4),180(2),169(1),195(1)	Daidzein
M3	5.49	C_16_H_11_O_5_	283.06119	283.06131	0.400	[M-H]^−^	126(100),195(11),239(7),268(5),69(3),283(2),62(2)	Glycitein
M4	9.89	C_16_H_11_O_5_	283.06119	283.06128	0.294	[M-H]^−^	112(100),283(48),195(23),239(21),178(17),268(11),184(10),240(8)	Calycosin
M5	7.64	C_20_H_17_O_9_	401.08777	401.08817	0.909	[M-H]^−^	253(100),241(50),197(42),223(2),122(5),136(4),116(9),161(5),91(3),107(3)	Arabinylation
M6	3.5	C_21_H_21_O_10_	433.11407	433.11346	−1.293	[M-H]^−^	116(100),272(37),228(15),117(6),153(2),66(2),165(1),109(1)	Carbonyl hydrogenation
M7	4.26	C_22_H_21_O_11_	461.10887	461.10886	−0.162	[M-H]^−^	180(100),109(99),300(12),147(5),87(4),151(1),97(1),163(1),241(1)	Hydroxylation and methylation
M8	0.96	C_20_H_21_O_9_	405.11802	405.11853	1.287	[M + H]^+^	336(100),227(73),289(51),201(43),215(35),69(30)	Decarbonylation
M9	9.20	C_31_H_36_O_17_N_3_S	754.17602	754.1803	4.846	[M + H]^+^	177(100),284(10),463(4),313(3),163(3),547(2),364(2),285(2)	Oxidation and GSH binding reaction
M10	4.26	C_13_H_5_O_5_	241.01435	241.01471	1.923	[M-H]^−^	197(100),241(42),141(38),109(3),105(1),178(1),175(1),73(1),126(1),121(1)	N-de-ethylation
	4.26	C_13_H_7_O_5_	243.02890	243.02963	3.416	[M + H]^+^	86(100),197(16),195(15),141(2),155(1),96(1)	
M11	9.63	C_14_H_9_O_4_	241.05065	241.05078	0.614	[M-H]^−^	123(100),108(19),199(12),147(10),117(7),69(7)	Decarbonylation
M12	12.50	C_15_H_11_O_5_	271.06125	271.06122	0.086	[M-H]^−^	209(100),205(7),117(7),233(6),203(5),180(5),68(3),93(3)	Carbonyl hydrogenation
M13	9.85	C_16_H_11_O_5_	283.06155	283.06131	0.400	[M-H]^−^	126(100),217(6),68(4),151(3),84(3),177(2),132(2)	Methylation
M14	4.43	C_14_H7O_7_	287.01965	287.0184	−4.619	[M-H]^−^	172(100),173(9),192(2),169(2),137(2),123(2),152(1),179(1)	Demethylation and dihydroxylation
M15	9.81	C_16_H_11_O_6_	299.05605	299.05618	0.230	[M-H]^−^	145(100),117(14),180(7),195(6),177(6),146(4),109(3)	Hydroxylation and methylation
M16	4.36	C_17_H_12_O_6_N	326.06705	326.06738	1.134	[M-H]^−^	116(100),74(18),171(11),326(10),226(3),125(2),98(1)	Glycine binding reaction
M17	3.15	C_8_H_5_O_5_	181.01330	181.01382	3.703	[M + H]^+^	135(100),109(31),125(25),56(13),137(6),139(3),73(3)	Debenzylation
M18	15.21	C_25_H_28_O_12_N_3_S	594.13890	594.13318	4.903	[M + H]^+^	89(100),109(9),177(8),137(6),125(2),153(1),287(1)	Epoxidation and GSH binding
M19	9.38	C_27_H_27_O_17_	623.12430	623.12189	−3.828	[M + H]^+^	133(100),177(26),195(5),221(4),269(2),287(1),248(1)	Diglucuronidation
M20	8.58	C_15_H_5_O_4_	249.01923	249.01865	−2.739	[M-H]^−^	176(100),117(6),183(4),134(3),89(3),107(3),67(2)	Dehydrogenation
M21	10.66	C_15_H_11_O_5_	271.06123	271.06122	0.086	[M-H]^−^	209(100),271(49),233(6),203(5),180(5),167(4),93(4),68(3)	Hydration
M22	9.92	C_16_H_11_O_5_	283.06113	283.06192	2.555	[M-H]^−^	126(100),195(12),179(8),139(6),165(4),167(4),151(3),93(2)	Hydroxylation and methylation
M23	7.62	C_15_H_9_O_6_	285.04043	285.04044	−0.075	[M-H]^−^	285(100),229(10),185(4),151(4),243(2),139(2),59(1)	Dihydroxylation
M24	0.91	C_20_H_16_O_6_NS	398.07043	398.06812	−4.681	[M-H]^−^	74(100),93(11),135(7),164(5),145(5),236(4),261(3),310(3)	Acetylcysteine binding reaction
M25	1.03	C_14_H_11_O_3_	227.07029	227.07083	2.463	[M + H]^+^	227(100),135(14),163(4),211(3),195(2),95(2),161(1)	Decarbonylation
M26	10.44	C_16_H_13_O_4_	269.08119	269.0798	−3.848	[M + H]^+^	269(100),213(14),238(2),181(2),163(1),108(1),93(1)	Methylation
M27	13.50	C_19_H_15_O_9_	387.07226	387.07037	−4.612	[M-H]^−^	89(100),297(1),107(1),161(1),91(1),65(1)	N-de-ethylation
M28	6.95	C_21_H_15_O_9_	411.07206	411.07227	0.279	[M-H]^−^	269(100),133(4),91(3),159(2),141(2),109(2),67(1)	Dehydrogenation
M29	14.33	C_23_H_22_O_10_N	472.12496	472.12753	4.773	[M-H]^−^	205(100),473(94),457(17),350(3),124(3),178(3),98(3)	Glycine binding reaction
M30	7.54	C_15_H_9_O_6_	285.04039	285.04044	−0.075	[M-H]^−^	285(100),177(6),107(5),255(4),151(4),83(3),268(2),243(2),133(1)	Demethylation and hydroxylation
M31	11.24	C_16_H_9_O_6_	297.04039	297.04031	−0.509	[M-H]^−^	183(100),269(38),117(7),93(5),211(4),255(3),205(3),179(3)	Hydroxylation and dehydrogenation
M32	9.78	C_16_H_13_O_6_	301.07179	301.07175	−0.038	[M-H]^−^	165(100),301(28),93(8),195(2),235(1),135(1),67(1)	Hydration
M33	9.60	C_17_H_13_O_6_	313.07169	313.0719	0.443	[M-H]^−^	313(100),167(18),149(4),271(3),191(3),123(3),220(2)	Hydroxylation and methylation
	9.60	C_17_H_15_O_6_	315.08625	315.08502	−4.109	[M + H]^+^	283(100),213(4),219(1),181(1),169(1),149(1),187(1)	
M34	6.20	C_18_H_14_O_6_N	340.08269	340.08401	3.968	[M-H]^−^	130(100),193(79),98(20),165(18),340(10),128(5),151(3)	Glycine binding reaction
M35	14.73	C_26_H_24_O_11_N_3_S	586.11369	586.10986	4.968	[M-H]^−^	83(100),233(30),368(13),410(5),215(4),586(2),177(1),139(1)	GSH binding and hydrogenation
M36	10.37	C_17_H_18_O_4_N	300.12307	300.12167	−4.547	[M + H]^+^	156(100),300(82),198(50),282(9),227(4),151(3),98(2)	Glycine binding reaction
M37	9.02	C_14_H_11_O_4_	243.06622	243.0661	3.763	[M-H]^−^	243(100),109(96),93(12),149(9),205(6),67(4),179(2)	Demethylation and hydroxylation
M38	0.91	C_15_H_13_O_6_S	321.04382	321.04269	−0.141	[M-H]^−^	135(100),177(7),121(4),321(4),149(3),225(2),147(2),171(1)	Sulfation
M39	3.77	C_21_H_23_O_8_	403.13982	403.1362	−4.693	[M-H]^−^	130(100),241(84),109(5),285(2),223(2),121(2)	Glucose binding reaction
M40	13.50	C_19_H_15_O_9_	387.07207	387.07037	−4.612	[M-H]^−^	89(100),313(1),309(1),297(1),201(1),180(1)	Hydroxymethyl loss and demethylation
M41	4.47	C_23_H_23_O_12_	491.11937	491.11911	−0.793	[M-H]^−^	127(100),130(37),180(32),193(20),461(16),223(11),205(11)	Dihydroxylation and dimethylation
M42	12.41	C_15_H_11_O_5_	271.06115	271.06122	0.086	[M-H]^−^	209(100),253(75),271(49),117(7),205(7),233(6),203(5),167(4)	Decarbonylation, hydroxylation and methylation
M43	8.58	C_14_H_9_O_5_	257.04555	257.04538	−0.648	[M-H]^−^	93(100),135(68),153(47),220(8),219(8),109(7),69(2)	Carbonyl hydrogenation and demethylation
M44	9.78	C_16_H_13_O_6_	301.07175	301.07175	−0.038	[M-H]^−^	165(100),269(33),301(28),137(10),109(6),151(5),121(5)	Carbonyl hydrogenation, hydroxylation and methylation
M45	4.37	C_16_H_9_O_5_	281.04543	281.04517	−1.340	[M-H]^−^	116(100),120(23),164(5),281(3),136(3),109(2)	Hydroxylation and methylation, hydroxylation and dehydration

**M0** exhibited the theoretical [M-H]^−^ ion at *m/z* 431.09837 (C_21_H_20_O_10_, −0.881 ppm) with a retention time of 5.10 min. Meanwhile, the fragment ion at *m/z* 91 ([M-H-C_15_H_16_O_9_]^−^) indicated the loss of B-ring, and the DPI at *m/z* 269 ([M-H-Glc]^−^) indicated that the glucose group at 7 position was removed. Furthermore, the fragment ions were compared with the genistin standard, and it was found that there was a high degree of coincidence. Therefore, **M0** was identified as genistin. **M1** was eluted at 9.46 min, and afforded [M-H]^−^ ion at *m/z* 269.04554 with the formula of C_15_H_9_O_5_. It was 162 Da less than that of genistin (*m/z* 431), suggesting that it might be genistein. In its MS^2^ spectrum, the DPIs at *m/z* 107 [(M-H-C_9_H_8_O_3_)^−^] and *m/z* 201 [(M-H-C_4_H_6_O)^−^] were generated due to RDA fragmentation. Meanwhile, compared with genistein reference substance, the fragment ions at *m/z* 159, *m/z* 133, and *m/z* 107 were consistent with genistein fragment ions. Therefore, **M1** was inferred to be genistein. **M2** could generate the [M-H]^−^ ion at *m/z* 253.05063, and was 162 Da less than that of daidzin (*m/z* 415). Meanwhile, its cleavage behavior was highly similar to daidzein. Consequently, **M2** was inferred to be daidzein.

**M3** yielded the [M-H]^−^ ion at *m/z* 283.06119 with the formula of C_16_H_11_O_5_. In its MS^2^ spectrum, the fragment ions at *m/z* 195 [M-H-C_3_H_3_O_3_]^−^, *m/z* 69 [M-H-C_12_H_10_O_4_]^−^, *m/z* 239 [M-H-C_2_H_4_O]^−^ were produced by the fragmentation of the quasi-molecular ion *m/z* 283. According to the literature, **M3** was identified as glycitein (Wu et al., 2004). **M4** exhibited the theoretical [M-H]^−^ ion at *m/z* 283.06119 (C_21_H_20_O_10_, 0.294 ppm) with a retention time of 9.89 min. In the MS^2^ spectrum, the B ring underwent RDA reaction to generate the fragment ion at *m/z* 184 [M-H-C_5_H_8_O_2_]^−^. Meanwhile, the fragment ion at *m/z* 239 indicated that the A ring was opened and one oxygen was removed. Finally, compared with the secondary fragments of calycosin standard, it was confirmed that **M4** was calycosin. **M5** was eluted at 7.64 min and produced the theoretical [M-H]^−^ ion at *m/z* 401.08777 (C_20_H_17_O_9_, 0.909 ppm), which was 132 Da higher than that of genistein. **M5** may be speculated to be the arabinylation product of genistein. In the MS^2^ spectra, the DPIs at *m/z* 223 [M-H-C_5_H_8_O_4_-CH_2_O_2_]^−^, *m/z* 253 [M-H-C_5_H_10_O_5_]^−^, and *m/z* 241 [M-H-C_7_H_10_O_6_]^−^ confirmed the theory above. Consequently, **M5** was identified as an arabinylation product of genistein.

**M6** showed the [M-H]^−^ ions at *m/z* 433.11407 (C_21_H_21_O_10_, mass error<5 ppm). The DPIs at *m/z* 153 [M-H-Glc-C_8_H_4_O]^−^, *m/z* 117 [M-H-Glc-C_7_H_6_O_4_]^−^, and *m/z* 165 [M-H-Glc-C_6_H_6_O_2_]^−^ gave evidence that **M6** was identified as carbonyl hydrogenation product of genistin. **M7** showed the theoretical [M-H]^−^ ions at *m/z* 461.10887 (C_22_H_21_O_11_, mass error<5 ppm) with 30 Da more massive than genistin. In its MS^2^ spectrum, the fragment ions at *m/z* 180 [M-H-Glc-C_7_H_8_O_2_]^−^, *m/z* 151 [M-H-Glc-C_9_H_10_O_2_]^−^ and *m/z* 87 [M-H-C_18_H_20_O_9_]^−^ proved that the reaction occurred at the 5′ position of the B ring. Consequently, **M7** was inferred to be the hydroxylation and methylation product of genistin. **M8** yielded the [M + H]^+^ ion at *m/z* 405.11802 with formula of C_20_H_21_O_9_. It was presumed to be a decarbonylation product of genistin. In its MS^2^ spectrum, the characteristic fragment ion at *m/z* 69 [M + H-C_16_H_16_O_8_]^+^ was produced by RDA rearrangement that occurred on the 3′ and 6′ positions of B-ring. Meanwhile, the fragment ions at *m/z* 227 [M + H-Glc-OH]^+^, *m/z* 289 [M + H-C_8_H_8_O]^+^, *m/z* 215 [M + H-Glc-2OH]^+^ confirmed that the decarbonylation reaction occurred at the 1 position of the C ring. Therefore, **M8** was identified as a decarbonylation product of genistin.

**M9** was eluted at 9.20 min and showed its [M + H]^+^ ion at *m/z* 754.17602 (C_31_H_36_O_17_N_3_S, mass error<5 ppm). It was 321 Da more than genistin, indicating that oxidation and glutathione (GSH) binding reactions may have occurred. In the ESI-MS/MS spectrum, the DPIs at *m/z* 547 [M + H-C_6_H_10_N_2_O_4_S]^+^, *m/z* 364 [M + H-C_8_H_9_O_4_N]^+^, *m/z* 313 [M + H-C_3_O]^+^ explained that **M9** was the product of oxidation and GSH binding reaction of genistin. The retention time of **M10** in the negative ion mode was 4.26 min, and the chemical formula was C_13_H_5_O_5_, with an error within 5 ppm. In its MS^2^ spectrum, it generated DPIs at *m/z* 178 [M-H-C_4_H_2_O]^−^, *m/z* 109 [M-H-C_7_H_4_O_3_]^−^, and *m/z* 105 [M-H-C_7_H_6_O_3_]^−^. Therefore, **M10** could be identified as a deethylation product of genistein. **M11** showed the theoretical [M-H]^−^ ions at *m/z* 241.05064 (C_14_H_9_O_4_, mass error<5 ppm) with 28 Da less massive than genistein. In its MS^2^ spectrum, the RDA reaction of the C ring generated fragment ions at *m/z* 117 and *m/z* 123, indicating that the decarbonylation reaction occurred at the 1 position of the C ring. Meanwhile, the above inference was also confirmed by fragment ions at *m/z* 199 [M-H-C_2_H_6_O]^−^ and *m/z* 108 [M-H-C_8_H_8_O_2_]^−^. Consequently, **M11** was identified as a decarbonylated product of genistein.

**M12** showed the theoretical [M-H]^−^ ions at *m/z* 271.06124 (C_15_H_11_O_5_, mass error<5 ppm) with 2 Da more massive than genistein. In its MS^2^ spectrum, the DPIs at *m/z* 233 [M-H-C_2_H_4_O]^−^ and *m/z* 203 were generated by B ring cleavage. Meanwhile, the fragment ion at *m/z* 117 [M-H-C_7_H_5_O_4_]^−^ was generated by the RDA reaction of the C ring, which proved that it occurred in the 1 position of the C ring. Therefore, **M12** was inferred to be a carbonyl hydrogenation product of genistein. **M13** showed the theoretical [M-H]^−^ ions at *m/z* 283.06154 (C_16_H_11_O_5_, mass error<5 ppm) with 14 Da more massive than genistein. In its MS^2^ spectrum, the B ring was removed to produce the fragment ion at *m/z* 177, which verified that the methylation reaction occurred on the B ring. At the same time, the generation of fragment ion at *m/z* 217 proved that the reaction occurs on the 4′-OH of the B ring. Therefore, **M13** was speculated to be the product of the methylation reaction of genistein. **M14** showed the theoretical [M-H]^−^ ions at *m/z* 287.01964 (C_14_H_7_O_7_, mass error<5 ppm) with 18 Da more massive than genistein. In its MS^2^ spectrum, the DPIs at *m/z* 152 [M-H-C_7_H_6_O_3_]^−^, *m/z* 123 [M-H-C_8_H_6_O_4_]^−^, and *m/z* 179 [M-H-C_6_H_6_O_2_]^−^ indicated that **M14** was identified as a demethylation and dihydroxylation product of genistein.

**M15** showed the theoretical [M-H]^−^ ions at *m/z* 299.05604 (C_16_H_11_O_6_, mass error<5 ppm) with 30 Da more massive than genistein. In its MS^2^ spectrum, the fragment ion at *m/z* 177 [M-H-C_7_H_8_O_2_]^−^ was generated by the removal of the B ring, which verified that the reaction occurred on the B ring. The above inference was also confirmed by the fragment ions at *m/z* 195 [M-H-C_6_H_6_O_2_]^−^ and *m/z*145 [M-H-C_6_H_6_O_2_-H_2_O_3_]^−^ produced by the cleavage of the C ring. Therefore, **M15** was identified as the hydroxylation and methylation product of genistein. **M16** showed the theoretical [M-H]^−^ ions at *m/z* 326.06704 (C_17_H_12_O_6_N, mass error<5 ppm) with 57 Da more massive than genistein. In its MS^2^ spectrum, the fragment ions at *m/z* 226 and *m/z* 99 were generated by breaking the B ring, which confirmed that the reaction occurred at the 4′ position of the B ring. At the same time, the loss of glycine group produced the fragment ion *m/z* 74, which also indicated the existence of the group. Therefore, **M16** was identified as a glycine-binding product of genistein. **M17** was eluted at 3.15 min and showed its [M + H]^+^ ion at *m/z* 181.01329 (C_8_H_5_O_5_, mass error<5 ppm). It was 90 Da less than genistein, indicating that a debenzyl reaction may have occurred. In the ESI-MS/MS spectrum, the DPIs at *m/z* 137 [M + H-CH_2_O_2_]^+^, *m/z* 125 [M + H-C_2_H_2_O_2_]^+^, and *m/z* 109 [M + H-C_2_H_2_O_3_]^+^ gave evidence that **M17** was identified as a debenzyl product of genistein.

**M18** was eluted at 15.21 min and showed its [M + H]^+^ ion at *m/z* 594.13889 (C_25_H_28_O_12_N_3_S, mass error<5 ppm). It was 323 Da more than genistein, indicating that the epoxy and GSH binding reaction may have occurred. The DPIs at *m/z* 287 [M + H-C_10_H_17_O_5_N_3_S]^+^, *m/z* 177 [M + H-C_16_H_23_O_8_N_3_S]^+^, and *m/z* 109 [M + H-C_19_H_23_O_10_N_3_S]^+^ gave evidence that **M18** was identified as the epoxy and GSH binding reaction product of genistein. **M19** was eluted at 9.38 min and showed its [M + H]^+^ ion at *m/z* 623.12429 (C_27_H_27_O_17_, mass error<5 ppm). It was 352 Da more than genistein, indicating that a diglucuronidation reaction may have occurred. In the ESI-MS/MS spectrum, the fragmentation of the B ring produced fragment ions at *m/z* 248 and *m/z* 177, which verified the existence of the glucuronic acid group and one of the groups was connected to the 4′-OH of the B ring. Meanwhile, the fragment ion at *m/z* 287 indicated that another glucuronic acid group was attached to the OH of the A ring. Therefore, **M19** was identified as a diglucuronidation product of genistein. The retention time of **M20** in the negative ion mode was 8.58 min, and the chemical formula was C_15_H_5_O_4_, with an error within 5 ppm. In its MS^2^ spectrum, it generated DPIs at *m/z* 183 [M-H-C_4_H_4_O]^−^, *m/z* 117 [M-H-C_7_H_4_O_3_]^−^, and *m/z* 107 [M-H-C_9_H_6_O_2_]^−^. Therefore, **M20** could be identified as a dehydrogenation product of daidzein.

**M21** could generate the [M-H]^−^ ion at *m/z* 271.06123, and was 18 Da more than that of daidzein. Meanwhile, the fragment ion at *m/z* 233 was produced by the cleavage of B ring and *m/z* 167 was generated by the cleavage of the C ring, indicating that the reaction should occur at the 3 position of the C ring. Thus, **M21** was presumed to be the hydration product of daidzein. **M22** could generate the [M-H]^−^ ion at *m/z* 283.06113, and was 30 Da more than that of daidzein. Meanwhile, the RDA reaction of the C ring produced the fragment ion at *m/z* 151 [M-H-C_7_H_8_O_3_]^−^ and the fragment ions at *m/z* 195 and *m/z* 179 produced by the cleavage of the C ring together proved that the reaction can only occur on the B ring. Therefore, **M22** was identified as the hydroxylation and methylation product of daidzein. **M23** was eluted at 7.62 min and showed its [M-H]^−^ ion at *m/z* 285.04043 (C_15_H_9_O_6_, mass error<5 ppm). It was 32 Da more than daidzein, indicating that a dihydroxylation reaction may have occurred. In the ESI-MS/MS spectrum, fragmentation ions at *m/z* 229 and *m/z* 185 were generated by B ring cleavage, verifying that the reaction occurred on the B ring. Meanwhile, the fragment ion at *m/z* 239 generated by the RDA reaction of the C ring also confirmed the above speculation. Therefore, **M23** was identified as a dihydroxylation product of daidzein.

**M24** was eluted at 0.91 min and showed its [M-H]^−^ ion at *m/z* 398.07043 (C_20_H_16_O_6_NS, mass error<5 ppm). It was 145 Da more than daidzein, indicating that an acetylcysteine binding reaction may have occurred. The DPIs at *m/z* 310 [M-H-C_6_H_6_O]^−^, *m/z* 261 [M-H-C_7_H_6_O_3_]^−^, and *m/z* 145 [M-H-C_15_H_9_O_4_]^−^ gave evidence that **M24** was identified as the acetylcysteine binding product of daidzein. **M25** was eluted at 1.03 min and showed its [M + H]^+^ ion at *m/z* 227.07028 (C_14_H_11_O_3_, mass error<5 ppm). It was 28 Da less than daidzein, indicating that a decarbonylation reaction may have occurred. In its MS^2^ spectrum, the fragmentation ion at *m/z* 161 was generated by the B ring cleavage, indicating that the reaction may occur in the A and B rings. Correspondingly, the loss of the two OH in the A and B rings produced the fragment ion at *m/z* 195, confirming that the reaction occurred in the C ring. Therefore, **M25** was identified as the decarbonylation product of daidzein. **M26** was eluted at 10.44 min and showed its [M + H]^+^ ion at *m/z* 269.08118 (C_16_H_13_O_4_, mass error<5 ppm). It was 14 Da more than daidzein, indicating that a methylation reaction may have occurred. The DPIs at *m/z* 238 [M + H-CH_4_O]^+^, *m/z* 213 [M + H-C_3_H_6_O]^+^, and *m/z* 163 [M + H-C_7_H_8_O]^+^ gave evidence that **M26** was identified as the methylation product of daidzein.

**M27** was 28 Da less than daidzin, and it showed the [M-H]^−^ ion at *m/z* 387.07225 (C_19_H_15_O_9_, mass error<5 ppm). Meanwhile, the DPIs at *m/z* 297 [M-H-C_6_H_2_O]^−^ and *m/z* 161 [M-H-Glc-C_4_H_2_O]^−^ were observed. Therefore, **M27** was identified as a deethylation product of daidzin. **M28** was 4 Da less massive than daidzin, and they showed the [M-H]^−^ ion at *m/z* 411.07205 (C_21_H_15_O_9_, mass error<5 ppm). Meanwhile, the DPIs at *m/z* 159 [M-H-C_15_H_10_O_4_]^−^ and *m/z* 141 [M-H-Glc-C_4_H_6_]^−^ were observed. Therefore, **M28** was identified as a dehydrogenation product of daidzin. **M29** was eluted at 14.33 min and showed its [M-H]^−^ ion at *m/z* 472.12495 (C_23_H_22_O_10_N, mass error<5 ppm). It was 57 Da more than daidzin, indicating that a glycine binding reaction may have occurred. In its MS^2^ spectrum, the fragment ions at *m/z* 350 and *m/z* 99 were generated by B ring cleavage, verifying that the reaction occurred at the 4’position of the B-ring. Therefore, **M29** was identified as the glycine binding product of daidzin.

**M30** was 2 Da more massive than glycitein, and they showed the [M-H]^−^ ion at *m/z* 285.04039 (C_15_H_9_O_6_, mass error<5 ppm). Meanwhile, the DPIs at *m/z* 268 [M-H-OH]^−^, *m/z* 243 [M-H-C_2_H_4_O]^−^ and *m/z* 151 [M-H-C_8_H_8_O_2_]^−^ were observed. Therefore, **M30** was identified as a demethyl-hydroxylated product of glycitein. **M31** was eluted at 11.24 min and showed its [M-H]^−^ ion at *m/z* 297.04039 (C_16_H_9_O_6_, mass error<5 ppm). It was 14 Da more than glycitein, indicating that hydroxylation and dehydrogenation reactions may have occurred. In its MS^2^ spectrum, the production of fragment ions at *m/z* 269 [M-H-CH_2_O]^−^ and *m/z* 255 [M-H-CH_2_O_2_]^−^ indicated that the reaction occurred on OH. Meanwhile, the fragment ion at *m/z* 211 [M-H-C_3_H_4_O_3_]^−^ was produced to crack through the A ring, which verified that the reaction occurred on the OH of the A ring. Therefore, **M31** was identified as a hydroxylation and dehydrogenation product of glycitetin. **M32** was eluted at 9.78 min and showed its [M-H]^−^ ion at *m/z* 301.07179 (C_16_H_13_O_6_, mass error<5 ppm). It was 18 Da more than glycitein, indicating that hydration reaction may have occurred. In its MS^2^ spectrum, the fragment ion at *m/z* 165 was generated by the C ring cleavage, indicating that the reaction may occur on the C ring and the B ring. Simultaneously, the fragment ions at *m/z* 235 and *m/z* 67 were produced by the cleavage of the B ring, which confirmed that the reaction occurred at the 2 position of the C ring. Consequently, **M32** was identified as a hydration product of glycitein.

**M33** was 30 Da more massive than glycitein, and they showed the [M-H]^−^ ion at *m/z* 313.07169 (C_17_H_13_O_6_, mass error<5 ppm). Meanwhile, the DPIs at *m/z* 271 [M-H-C_2_H_4_O]^−^, *m/z* 220 [M-H-C_5_H_8_O_2_]^−^ and *m/z* 167 [M-H-C_9_H_10_O_2_]^−^ were observed. **M33** was identified as the hydroxylation and methylation product of glycitein. **M34** was 57 Da more massive than glycitein, and it showed the [M-H]^−^ ion at *m/z* 340.08269 (C_18_H_14_O_6_N, mass error<5 ppm). Meanwhile, the DPIs at *m/z* 193 [M-H-C_8_H_9_O_2_N]^−^, *m/z* 151 [M-H-C_10_H_10_O_4_]^−^ and *m/z* 128 [M-H-C_12_H_10_O_4_]^−^ indicated that the reaction can only occur on the OH of the B ring. Therefore, **M34** was identified as the glycine binding product of glycitein. **M35** was 303 Da more massive than glycitein, and it showed the [M-H]^−^ ion at *m/z* 586.11369 (C_26_H_24_O_11_N_3_S, mass error<5 ppm). In its MS^2^ spectrum, the generation of fragment ions at *m/z* 410 and *m/z* 368 indicated the loss of some GSH groups. Meanwhile, the generation of fragment ion at *m/z* 215 indicated that the GSH binding reaction occurred on the B ring. Therefore, **M35** was identified as a GSH-binding and hydrogenation product of glycitein.

**M36** was 57 Da more massive than equol, and they showed the [M + H]^+^ ion at *m/z* 300.12307 (C_17_H_18_O_4_N, mass error<5 ppm). Meanwhile, the DPIs at *m/z* 281 [M + H-OH]^+^, *m/z* 227 [M + H-C_2_H_5_O_2_N]^+^ and *m/z* 151 [M + H-C_9_H_10_O_2_]^+^ were observed. **M36** was identified as glycine-binding product of equol. **M37** exhibited the theoretical [M-H]^−^ ion at *m/z* 243.06621 (C_14_H_11_O_4_, 3.763 ppm) with a retention time of 9.02 min. In the MS^2^ spectrum, the fragmentation ions at *m/z* 179 and *m/z* 205 were generated by the B ring cracking, indicating that the reaction may occur in the C ring. Meanwhile, the fragment ion at *m/z* 109 was produced by the cracking of the C ring, verifying that the reaction occurred at positions 2 and 4 of the C ring. Consequently, **M37** was identified as the demethylation and hydroxylation product of equol. **M38** was 80 Da more massive than equol, and they showed the [M-H]^−^ ion at *m/z* 321.04381 (C_15_H_13_O_6_S, mass error<5 ppm). Meanwhile, the DPI at *m/z* 225 [M-H-H_2_O_4_S]^−^ was produced by the removal of the sulfated group and an OH. The cleavage of B ring and C ring produced the fragment ions at *m/z* 177 [M-H-C_4_H_4_O_4_S]^−^ and *m/z* 121 [M-H-C_8_H_10_O_4_S]^−^, respectively, which confirmed that the reaction occurred on the OH of B ring. Therefore, **M38** was identified as a sulfated product of equol.

**M39** was 162 Da more massive than equol, and it showed the [M-H]^−^ ion at *m/z* 403.13981 (C_21_H_23_O_8_, mass error<5 ppm). Meanwhile, the DPIs at *m/z* 285 [M-H-C_8_H_10_O]^−^, *m/z* 241 [M-H-Glc]^−^ and *m/z* 109 [M-H-Glc-C_9_H_12_O]^−^ were observed. Therefore, **M38** was identified as a glucose-binding product of equol. **M40** was eluted at 13.50 min and showed its [M-H]^−^ ion at *m/z* 387.07207 (C_19_H_15_O_9_, mass error<5 ppm). It was 44 Da more than genistin, indicating that hydroxymethyl loss and demethylation reaction may have occurred. In its MS^2^ spectrum, the DPI at *m/z* 313 [M-H-Glc-C_9_H_12_O]^−^ was generated by B ring loss, indicating that the hydroxymethyl loss reaction occurred in the glucose group. Meanwhile, the DPIs at *m/z* 309 [M-H-C_5_H_4_O]^−^ and *m/z* 180 [M-H-C_12_H_8_O_4_]^−^ also confirmed the above view. Therefore, **M40** was identified as a hydroxymethyl loss and demethylation product of genistin. **M41** was 60 Da more massive than genistin, and it showed the [M-H]^−^ ion at *m/z* 491.11937 (C_23_H_23_O_12_, mass error<5 ppm). Meanwhile, the DPIs at *m/z* 461 [M-H-CH_4_O]^−^, *m/z* 193 [M-H-C_13_H_16_O_8_]^−^ and *m/z* 180 [M-H-C_7_H_6_O_4_]^−^ were observed. Therefore, **M41** was identified as two hydroxymethyl loss products of genistin.

**M42** was 2 Da more massive than genistein, and it showed the [M-H]^−^ ion at *m/z* 271.06114 (C_15_H_11_O_5_, mass error<5 ppm). Meanwhile, the DPIs at *m/z* 253 [M-H-H_2_O]^−^, *m/z* 233 [M-H-C_2_H_4_O]^−^ and *m/z* 167 [M-H-C_6_H_6_O_2_]^−^ were observed. **M42** was identified as a decarbonylation, hydroxylation, and methylation product of genistein. **M43** was 12 Da less than genistein, and it showed the [M-H]^−^ ion at *m/z* 257.04554 (C_14_H_9_O_5_, mass error<5 ppm). Meanwhile, the DPIs at *m/z* 220 [M-H-C_2_H_4_O]^−^, *m/z* 153 [M-H-C_6_H_6_O_2_]^−^ and *m/z* 109 [M-H-C_8_H_10_O_3_]^−^ were observed. **M43** was identified as a carbonyl hydrogenation and demethylation product of genistein. **M44** exhibited the theoretical [M-H]^−^ ion at *m/z* 301.07174 (C_16_H_13_O_6_, −0.038 ppm) with a retention time of 9.78 min. In the MS^2^ spectrum, the fragment ion at *m/z* 121 was generated by the removal of the B ring, indicating that the hydroxylation and methylation reaction occurred on the B ring. Meanwhile, the DPIs at *m/z* 269 [M-H-CH_4_O]^−^ and *m/z* 151 [M-H-C_7_H_6_O_3_]^−^ were observed. Therefore, **M44** was identified as a carbonyl hydrogenation, hydroxylation, and methylation product of genistein. **M45** was 28 Da more than daidzein, and it showed the [M-H]^−^ ion at *m/z* 281.04543 (C_16_H_9_O_5_, mass error<5 ppm). Meanwhile, the DPIs at *m/z* 164 [M-H-C_7_H_6_O_2_]^−^, *m/z* 136 [M-H-C_9_H_10_O_2_]^−^ and *m/z* 120 [M-H-C_9_H_8_O_3_]^−^ were observed. **M45** was identified as hydroxylation and methylation, hydroxylation, and dehydration product of daidzein.

### 3.4 16S rRNA gene-based amplicon sequencing and bioinformatic analysis

**FIGURE 3:**
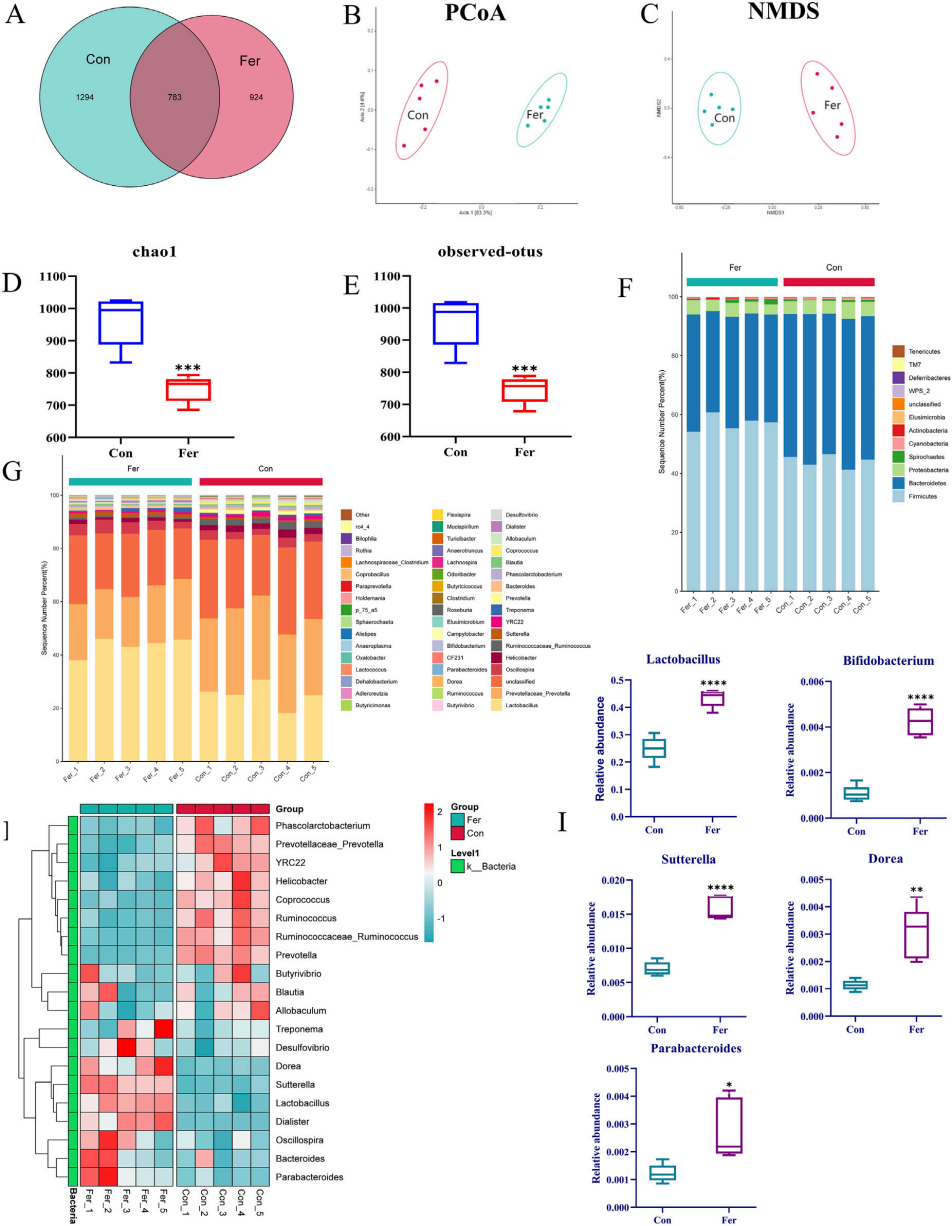
Analysis results of 16S rRNA in fecal fermentation of genistin. **(A)** Venn diagrams of bacterial communities in Con and Fer groups. **(B)** PCoA analysis among Con and Fer groups. **(C)** NMDS analysis among Con and 24 h groups. **(D)** Chao1 and **(E)** observed OTUs represent alpha-diversity indexes for each sample group. **(F,G)** Histograms of species distribution at phylum and genus levels for the two groups were shown by 16S rRNA sequencing (different colors represent different bacteria at phylum or genus levels). **(H)** Heat map of cluster analysis between two groups (Red and blue represent increased and decreased bacteria content, respectively). **(I)** The results of the level of 5 different dominant bacteria genera among the groups in 24 h (n = 5, data are presented as mean ± SD). ^*^ *p* < 0.05, ^**^ *p* < 0.01, ^***^ *p* < 0.001, ^****^ *p* < 0.0001.

In this study, 10 fecal samples from the control (Con) and fermentation (Fer) groups were analyzed by 16S rRNA sequencing. A total of 165,628 reads were generated by high-throughput sequencing, and a total of 3,001 species taxonomic OTUs were obtained by passing all quality filters with an identification threshold of 97%. Principal coordinate analysis (PCoA) and nonmetric multidimensional scaling analysis (NMDS) were used to describe beta diversity. PCoA (Figure 3B) and NMDS (Figure 3C) analyses revealed distinct clusters between the two groups, indicating significant community differences. The results of PCoA and NMDS showed that there was a significant separation trend in the Con and Fer groups, indicating that the intestinal microbial community was significantly different from the Con group after 24 h of biotransformation. The Venn diagram can be used to show the structural similarity and overlap of species in different groups of samples. There were 783 common species in the Con and Fer group, 924 unique species in the Fer group, and 1 294 unique species in the Con group (Figure 3A). This indicated that after 24 h of genistin fermentation, the intestinal microbial community of rats produced unique species. Furthermore, the results of chao1 and observed OTUs are shown in Figures 3D, E. It can be found that the bacterial community of the Con group was more diverse than that of the Fer group.

The results of species annotation analysis of the two groups at the phylum and genus levels are shown in Figures 3F, G. At the phylum level, *Firmicutes*, *Bacteroidetes*, and *Proteobacteria* were the most dominant phyla in intestinal bacteria of SD rats, accounting for the main relative abundance in all samples. Furthermore, compared with Con, the *Bacteroidetes* in the Fer group decreased significantly. *Firmicutes* and *Actinobacteria* increased significantly. In the whole genus composition, there were significant differences in 5 genera between the Con group and the Fer group (Figure 3I). Among them, the relative abundance of *Lactobacillus*, *Parabacteroides*, *Bifidobacterium*, *Sutterella,* and *Dorea* increased significantly after 24 h fermentation (*p* < 0.05). Of note, *Lactobacillus*, *Prevotella,* and *Bifidobacterium* account for a large proportion of the total genus composition. Therefore, this also provided a great reference value for the subsequent screening of five probiotics for fermentation verification. Meanwhile, 20 species of bacteria were screened in the whole genus for cluster analysis, and the results are shown in Figure 3H. The results showed that compared to the Con group, the flora of the Fer group was significantly different.

### 3.5 HPLC analysis of genistin fermentation by five probiotics *in vitro*

**FIGURE 4:**
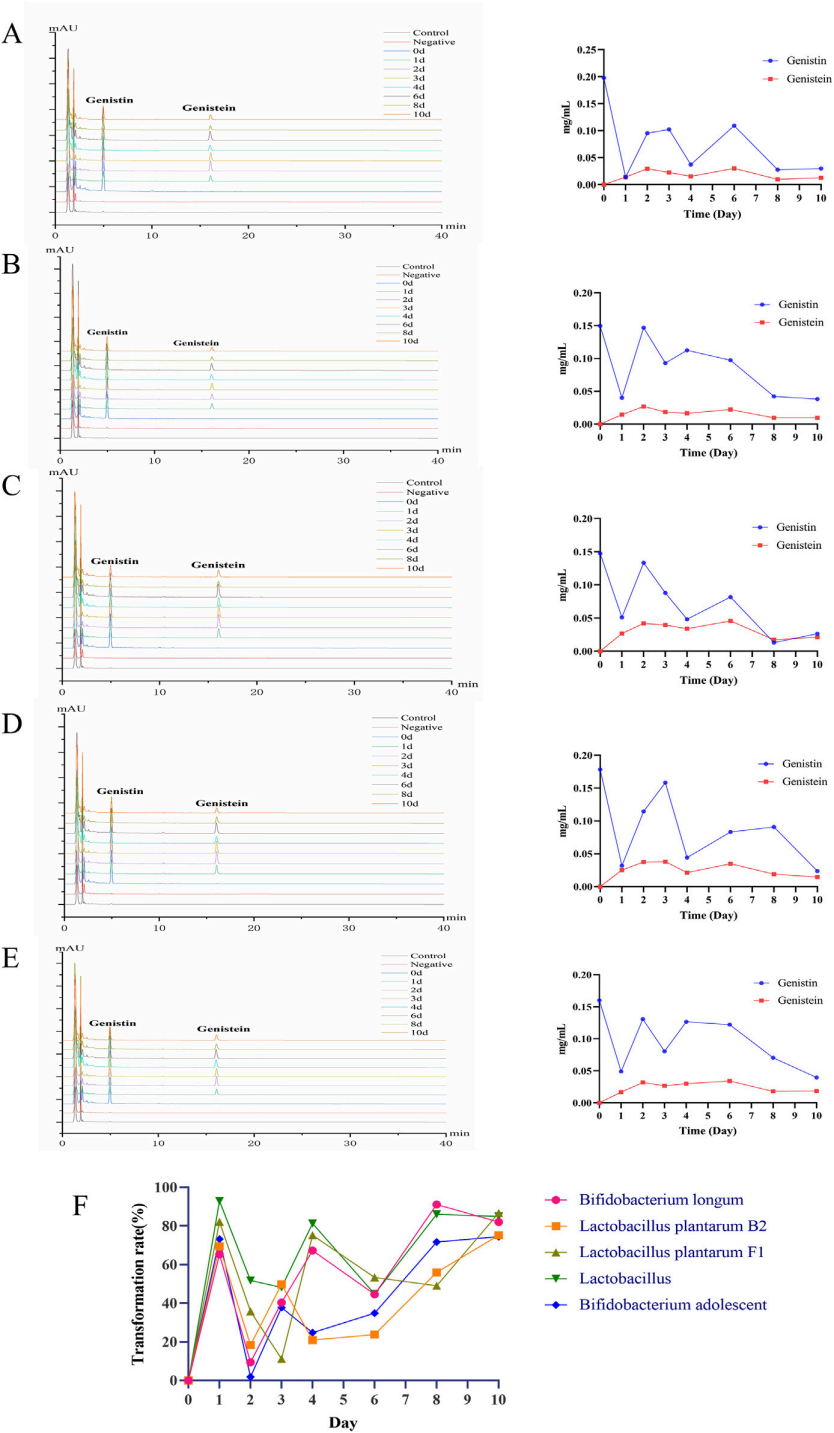
HPLC analysis of biotransformation products of five probiotics (left: the chromatogram of biotransformation products; right: the line chart showing the trend of biotransformation products). **(A)** *Lactobacillus*. **(B)** Bifidobacterium adolescent. **(C)** Bifidobacterium longum. **(D)** *Lactobacillus* plantarum F1. **(E)** *Lactobacillus* plantarum B2. **(F)** Bioconversion rate of fermentation.

The levels of genistin fermentation products by five probiotics (*Lactobacillus*, *Bifidobacterium longum*, *Bifidobacterium adolescent*, *L. plantarum F1,* and *L. plantarum B2*) at different time points were analyzed by HPLC. The results are shown in Figure 4. For *Bifidobacterium longum*, the level of genistin decreased from 0.1474 mg/mL to 0.0265 mg/mL within 10 days of fermentation, and the content of genistein increased from zero to 0.0213 mg/mL (Figure 4C). For *L. plantarum B2*, the content of genistin decreased from 0.1601 mg/mL to 0.0396 mg/mL within 10 days of fermentation, and the content of genistein increased from zero to 0.0184 mg/mL (Figure 4E). For *L. plantarum F1*, the content of genistin decreased from 0.1785 mg/mL to 0.0238 mg/mL within 10 days of fermentation, and the content of genistein increased from zero to 0.0145 mg/mL (Figure 4D). For *Lactobacillus*, the content of genistin decreased from 0.1978 mg/mL to 0.0298 mg/mL within 10 days of fermentation, and the content of genistein increased from zero to 0.0127 mg/mL (Figure 4A). For *Bifidobacterium adolescentis*, the content of genistin decreased from 0.1497 mg/mL to 0.0382 mg/mL within 10 days of fermentation, and the content of genistein increased from zero to 0.0096 mg/mL (Figure 4B).

Additionally, the bioconversion of genistin by the five probiotics was tortuous (Figure 4F). The average transformation rates of *Bifidobacterium longum*, *L. plantarum B2*, *L. plantarum F1*, *Lactobacillus* and *Bifidobacterium adolescentis* were 57.16%, 44.78%, 56.19%, 70.01%, and 45.54%, respectively. Apparently, the bioconversion rate of *Lactobacillus* was the highest, which may be related to the predominance of *Lactobacillus* at the genus level in fecal bacteria. Unfortunately, the HPLC results showed that genistin was not completely converted after 10 days of fermentation, which may be attributed to the incomplete enzymatic hydrolysis of genistin glycosidic bonds by β-glucosidase produced by a single strain cultured *in vitro*.

### 3.6 UHPLC-Q-Exactive Orbitrap MS analysis of five probiotics and genistin fermentation based on *in vitro* culture at different times

Based on the distribution characteristics of genistin metabolites, the metabolic results of fecal fermentation were verified by five probiotics cultured *in vitro*. The results are shown in Table 2. Among the 46 metabolites were fermented by fecal bacteria, 28, 32, 34, 21, and 36 metabolites were found in *Lactobacillus*, *Bifidobacterium longum*, *Bifidobacterium adolescentis*, *L. plantarum F1* and *L. plantarum B2*, respectively. Obviously, the reaction of fecal bacteria and genistin can produce many metabolites. However, the metabolites of different bacteria also had similarities and differences. Among them, there were 18 common metabolites among the five probiotics, such as **M0**, **M1**, **M2**, **M4**, **M7**, **M10**, **M13**, **M19**, **M22**, **M23**, **M30**, **M32**, **M33**, **M36**, **M39**, **M41**, **M44**, and **M45**. 9 metabolites were common to the four probiotics, such as **M12**, **M21**, **M26**, **M27**, **M37**, **M38**, **M40**, **M42**, and **M43**. Coincidentally, among the 46 metabolites, some metabolites only exist in the fermentation products of specific probiotics, such as **M3** in *Lactobacillus*, **M16** in *Bifidobacterium longum,* and **M5** in *Bifidobacterium adolescentis*. The production of these specific metabolites may be the main driving force for the biotransformation of specific bacteria. Correspondingly, **M8**, **M11**, **M17**, **M29,** and **M35** were not found in the fermentation metabolites of the five probiotics. This may be the result of the interaction between enzymes produced by other bacteria in feces and genistin.

**TABLE 2:** Distribution of metabolites from genistin faecal fermentation in five kinds of probiotics fermentation.

Peaks	*Lactobacillus*	*Bifidobacterium longum*	*Bifidobacterium adolescent*	*Lactobacillus plantarum F1*	*Lactobacillus plantarum B2*
M0	√	√	√	√	√
M1	√	√	√	√	√
M2	√	√	√	√	√
M3	√	×	×	×	×
M4	√	√	√	√	√
M5	×	×	√	×	×
M6	√	×	×	×	√
M7	√	√	√	√	√
M8	×	×	×	×	×
M9	×	×	√	×	√
M10	√	√	√	√	√
M11	×	×	×	×	×
M12	√	√	√	×	√
M13	√	√	√	√	√
M14	×	√	√	×	×
M15	×	√	×	×	√
M16	×	√	×	×	×
M17	×	×	×	×	×
M18	×	√	√	×	√
M19	√	√	√	√	√
M20	×	√	√	×	√
M21	√	√	√	×	√
M22	√	√	√	√	√
M23	√	√	√	√	√
M24	×	√	√	×	√
M25	√	√	×	×	√
M26	×	√	√	√	√
M27	√	×	√	√	√
M28	×	×	√	×	√
M29	×	×	×	×	×
M30	√	√	√	√	√
M31	×	×	√	×	√
M32	√	√	√	√	√
M33	√	√	√	√	√
M34	√	√	×	×	√
M35	×	×	×	×	×
M36	√	√	√	√	√
M37	√	√	√	×	√
M38	√	√	√	×	√
M39	√	√	√	√	√
M40	√	×	√	√	√
M41	√	√	√	√	√
M42	√	√	√	×	√
M43	×	√	√	√	√
M44	√	√	√	√	√
M45	√	√	√	√	√

√: Metabolites were present. ×: Metabolites were not present.

Simultaneously, the distribution of genistin fecal fermentation metabolites in five probiotics at different time points was analyzed. The results of fermentation at different time points are shown in Supplementary Tables S2–6. In *Lactobacillus*, a total of 6 metabolites were detected in 10 consecutive days of fermentation. Similarly, *Bifidobacterium longum*, *Bifidobacterium adolescentis*, *L. plantarum F1*, and *L. plantarum B2* also have 14, 10, 12, and 9 metabolites were identified at different time points. This may be the main metabolite of genistin fermented by five probiotics. Meanwhile, **M1** (genistein) was identified on 0 days in the *in vitro* fermentation of the five probiotics and most of the metabolites were identified on 0 days and 1 day, indicating that genistin fermentation can quickly produce secondary metabolites and intestinal bacteria have a strong degradation ability. It was worth noting that **M12** was produced at 0 days of fermentation. It disappeared during the first and second days of fermentation. Subsequently, it was reappeared in subsequent times. This may be that **M12** was produced at 0 days and degraded to other metabolites at 2 days. Finally, it was produced again under the action of intestinal flora. In this study, there were many similar cases, such as **M18**, **M21**, **M25**, and **M42**.

### 3.7 Absorption analysis based on the Caco-2 cell monolayer

**FIGURE 5:**
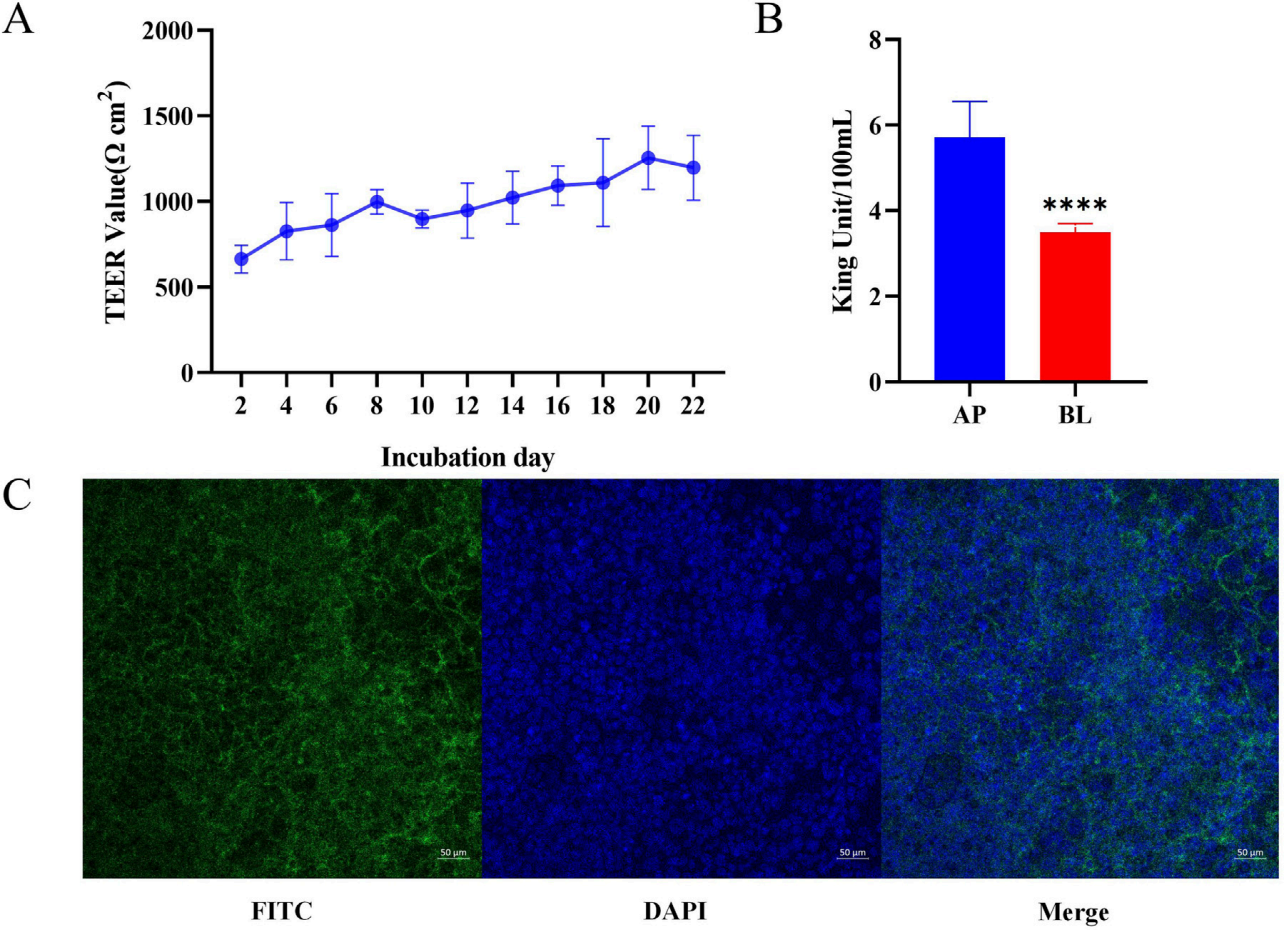
Verification of Caco-2 cell monolayer. **(A)** Determination of cell resistance for 21 consecutive days. **(B)** Alkaline phosphatase activity on day 21. **(C)** Validation of laser confocal single-layer film. ^*^ *p* < 0.05,^**^ *p* < 0.01, ^***^ *p* < 0.001, ^****^ *p* < 0.0001.

Cell transmembrane resistance, alkaline phosphatase activity, and laser confocal observation were three important indicators to determine whether the Caco-2 cell monolayer was successfully established (Noda et al., 2016). The transmembrane resistance value of the cell membrane is the most intuitive and simplest method to evaluate the integrity and tightness of the Caco-2 cell membrane. The higher the transmembrane resistance value, the more complete the Caco-2 cell membrane is, and the closer the connection between adjacent cells. The results of cell resistance measurement are shown in Figure 5A. On the second day after inoculation, cell resistance was measured to be 567.84 ΩCM^2^. On day16, cell resistance began to stabilize (1092.56 ΩCM^2^), indicating that Caco-2 cells had not only completely fused, but also began to differentiate. The cells began to tightly connect the structure and gradually expressed protein carriers and enzymes related to absorption and metabolism. On day22 of culture, cell resistance began to decline, which may be attributed to the cells beginning to appear laminated, resulting in some cell growth being inhibited. Consequently, the verification experiment of monolayer membrane absorption was performed on day21 after cell plating. Alkaline phosphatase is a marker enzyme for the polar differentiation of intestinal brush-border cells. When polar differentiation was completed, the alkaline phosphatase activity of the AP was significantly higher than that of the BL. Consequently, alkaline phosphatase activity was measured on the AP and BL on day 21 (Figure 5B). The results showed that the AP was 5.72 ± 0.8353 King Units, and the BL was 3.49 ± 0.2055 King Units. The alkaline phosphatase activity of the AP was significantly higher than that of the BL (*p* < 0.0001). This indicated that cell polarity differentiation was completed and could be used for monolayer absorption experiments. The distribution of nuclear and plasma membrane proteins in Caco-2 cells was determined by 4′,6-diamidino-2-phenylindole (DAPI) and FITC-phalloidin staining, respectively. Confocal laser scanning microscopy results showed that the integrity and tight connection of the Caco-2 cell membrane and nucleus were good, which met the requirements of the simulated small intestine for material absorption experiments (Figure 5C). The MTT experiment results are shown in Supplementary Figure S1. Different concentrations of genistin fermentation broth had no significant effect on the OD value. This indicated that genistin fermentation broth did not have reproductive toxicity to Caco-2 cells and was not dose dependent.

The absorption results of five probiotics based on the Caco-2 cell monolayer on the metabolites after genistin fermentation are shown in Table 3. Surprisingly, a total of 15 metabolites were detected on the AP and on the BL, indicating that there was no difference in the absorption of these metabolites after fermentation of genistin by five probiotics. Meanwhile, these metabolites may be the main form of absorption of genistin after biotransformation. Obviously, as an isoflavone glycoside, it has been reported that genistin is difficult to absorb by the human body as a prototype drug (Li et al., 2023). Fortunately, the results of the Caco-2 cell absorption experiments undoubtedly proved this view. Interestingly, these 14 metabolites could all enter the BL through the monolayer, indicating that the metabolites can be well absorbed by cells in the intestinal wall and enter the systemic circulation.

**TABLE 3:** Results of metabolite absorption in Caco-2 cells.

Peaks	*Lactobacillus*		*Bifidobacterium longum*		*Bifidobacterium adolescent*		*Lactobacillus plantarum F1*		*Lactobacillus plantarum B2*	
	AP	BL	AP	BL	AP	BL	AP	BL	AP	BL
M0	√	×	√	×	√	×	√	×	√	×
M1	√	√	√	√	√	√	√	√	√	√
M2	√	√	√	√	√	√	√	√	√	√
M7	√	√	√	√	√	√	√	√	√	√
M10	√	√	√	√	√	√	√	√	√	√
M19	√	√	√	√	√	√	√	√	√	√
M23	√	√	√	√	√	√	√	√	√	√
M25	√	√	√	√	√	√	√	√	√	√
M30	√	√	√	√	√	√	√	√	√	√
M33	√	√	√	√	√	√	√	√	√	√
M36	√	√	√	√	√	√	√	√	√	√
M37	√	√	√	√	√	√	√	√	√	√
M38	√	√	√	√	√	√	√	√	√	√
M39	√	√	√	√	√	√	√	√	√	√
M41	√	√	√	√	√	√	√	√	√	√

√: Metabolites were present. ×: Metabolites were not present.

## 4 Discussion

Studies have reported that the concentration of genistin in plasma is usually found in the range of 0.01–0.1 μM, which was significantly lower than the commonly reported IC_50_ or EC_50_ value of 5–50 μM (Setchell et al., 2001; Liu and Hu, 2002; Yuan et al., 2012). Consequently, these results revealed the non-absorptive of genistin. Meanwhile, the fermentation technology of traditional Chinese medicine can be used to catalyze the decomposition of drugs under enzymes and microorganisms, and obtained small molecular compounds. Our previous studies have shown that the intestinal microbiota plays an irreplaceable role in the decomposition of genistin into small-molecule metabolites or the production of new and unknown compounds during human fecal fermentation (Liu et al., 2023). In this study, the content of each component after fermentation was first determined by fermentation of genistin and fecal bacteria. The results showed that the genistin content decreased markedly, while the genistein content exceeded that of genistin after 24 h of fermentation. This result also confirmed that glycoside can be converted into aglycone by fermentation. Subsequently, the trace components after fermentation were qualitatively analyzed by UHPLC-Q-Exactive Orbitrap MS. A total of 46 metabolites were identified, involving the products of hydroxylation and methylation, glycine binding, decarbonylation, hydration, GSH binding, and other metabolic reaction. Meanwhile, a metabolic pathway network was constructed using five prototype drugs of genistin, genistein, daidzin, daidzein, and glycitein (Figure 2). The network analysis showed that deglycosylation, glycine binding, and diglucuronic acid binding were the main metabolic reaction products of genistin in the feces of Sprague-Dawley (SD) rats. It should be noted that genistin metabolites after fermentation with fecal flora have not been described in previous studies on the *in vivo* metabolic behavior of genistin, such as glycine binding, acetylcysteine binding, GSH binding and other metabolic reactions. Therefore, these results provided a new direction for the follow-up study of genistin. Moreover, the above metabolites are also essential to reveal the biotransformation reaction of genistin *in vivo*.

Furthermore, 16S rRNA sequencing was used to explore the role of intestinal flora in the transformation of genistin. The results showed that the number of bacteria changed significantly after fermentation compared with the Con group, and 924 unique bacteria were produced. At the phylum level, the relative abundances of *Firmicutes*, *Bacteroidetes*, and *Proteobacteria* represented the majority of the microbial phylum. After fermentation, the relative abundance of *Firmicutes* was increased significantly and the relative abundance of *Bacteroidetes* was decreased significantly, indicating that *Firmicutes* and *Bacteroidetes* were associated with the transformation of genistin. Studies have shown that the proportion of *Firmicutes*: *Bacteroidetes* is associated with susceptibility to disease status (Marín et al., 2015). The members of *Bacteroidetes* are the main organisms involved in carbohydrate metabolism, which is accomplished by expressing enzymes such as glycosyltransferases, glycoside hydrolases, and polysaccharide lyases (Cantarel et al., 2012). Meanwhile, studies have shown that after ingestion of isoflavones, the sugar part is separated from the phenolic trunk in the small intestine and absorbed here (Day et al., 2000). Enzymes such as lactase-phenylpropanol hydrolase (LPH) (on the intestinal cell membrane) hydrolyze glycosylated flavonoids, and then the aglycone diffuses into the epithelial cells through passive diffusion (Day et al., 1998). Therefore, the reduction of *Bacteroidetes* may promote the cleavage and biotransformation of genistin glycosidic bonds. For the genus level, the relative abundance of *Lactobacillus*, *Prevotella,* and *Bifidobacterium* amounted to 50% of the total bacteria. Interestingly, as a component of the *Firmicutes*, it is speculated that the increase in the content of *Lactobacillus* after fermentation may lead to the change in the content of *Firmicutes*, which also confirmed that *Lactobacillus* may be the key to promoting genistin conversion. *Lactobacillus* is considered to be related to the promotion of cholesterol metabolism, immune regulation, and antioxidants (Tannock, 2002). In addition, it has been reported that rhamnosidase from *Lactobacillus acidophilus* can effectively degrade flavonoids and flavonol glycosides, such as hesperidin, naringin and rutin (Beekwilder et al., 2009). As a part of *Actinobacteria*, *s*tudies have reported that *bifidobacterium* has the functions of protecting the liver, preventing and treating cardiovascular diseases, and improving lactose digestion (Slattery et al., 2019). *Bifidobacterium*, as a glycolytic bacterium, has been shown to have the ability to ferment glucose, galactose and fructose (Palframan et al., 2003). So far, more than 50 different bifidobacterial glycosidases have been identified. This further emphasizes that the members of the genus specialize in carbohydrate metabolism (Cronin et al., 2011). In addition, studies have reported that *bifidobacteria* metabolize astragaloside IV very quickly and produce brachyoside B within 24 h (Takeuchi et al., 2022). Consequently, these two bacteria may be the key to promoting the biotransformation of genistin. The quantitative results of genistin fermentation by five probiotics showed that the change of genistin content was tortuous and the content of genistin reached the lowest level on the tenth day of fermentation. Studies have reported that probiotics such as *Lactobacillus* and *bifidobacteria* can decompose or convert flavonoids into new active ingredients under the action of enzymes, thereby improving the biological activity of natural medicines (Yang et al., 2023). Notably, genistein was produced on the first day of fermentation, which also verified the research conclusions in the literature. The results of HPLC showed that the conversion rate of genistin by the five probiotics was higher. Furthermore, the change of genistin level was unstable at different time of biotransformation, revealing the resynthesis of genistin. *Bifidobacterium longum* was a Gram-positive anaerobic bacterium and the most abundant *bifidobacteria* in the gastrointestinal tract of healthy infants and adults (Roberts et al., 2023). *Bifidobacterium adolescentis* metabolizes hexose sugars through the ‘bifid shunt’ with fructose-6-phosphoketolase as the key enzyme generating ATP and mainly acetate and lactate (Pokusaeva et al., 2011). *Bifidobacterium longum* was the most abundant and *Bifidobacterium adolescenti*s was the least abundant in the conversion of genistin to genistein. This may be attributed to the high content of *Bifidobacterium longum* in *Bifidobacterium*, which promotes the occurrence of biotransformation reactions. Unfortunately, HPLC results showed that only genistein was produced in the bioconversion reaction, while the two new metabolites in the fecal fermentation were not found. This may be attributed to the production of two metabolites derived from the hydrolysis of genistin by other bacteria. Dominant probiotics have an important effect on drug metabolism in intestinal flora (Wieërs et al., 2020). The UHPLC-Q-Exactive Orbitrap MS results showed a high degree of similarity between numerous metabolites by *in vitro* fecal fermentation and fermentation of five probiotics. Interestingly, the metabolic profiles of five probiotics were significantly different, indicating that different probiotics may secrete different bioenzymes to generate different metabolites by transforming genistin. The metabolites produced by five probiotics had been generated at 0 h and 24 h, the results verified that probiotics were able to quickly eliminate genistin. Coincidentally, some metabolites were also disappeared during the transformation. This results fully showed that these metabolites might be transformed into other products with series of metabolic reactions.

According to the previous studies on the transformation of genistein by intestinal microorganisms and probiotics, it was found that *in vitro* probiotic fermentation can be used to verify the products of intestinal microbial fermentation. However, whether such transformed metabolites can be rapidly absorbed has not been reported. Consequently, it was necessary to verify the absorption of the fermented product of fecal bacteria through the Caco-2 cell monolayer. The results showed that a total of 14 metabolites were detected on the BL, involving the reaction products of genistein, daidzein, hydroxylation and methylation, de-ethylation, diglucuronic acid binding, dihydroxylation, decarbonylation, demethylation and hydroxylation, glycine binding, sulfation, glucose binding. In biotransformation reactions, once the final derivative or sugar was absorbed (in the small intestine or colon), it undergoes a certain degree of phase II metabolism at the intestinal cell level, such as methylation, sulfonation and glucuronidation (Monagas et al., 2010). Subsequently, these products were bound and transported to the blood again after more phase II metabolism by the intestinal flora until they are secreted in the urine (Selma et al., 2009). The bioavailability of isoflavones requires the conversion of glycosides into biologically active aglycones through the action of small intestinal bacteria (*Lactobacillus*, *Bifidobacterium*) β-glucosidases (Setchell et al., 2002). Subsequently, these aglycones were absorbed into the peripheral circulation. Studies have reported that some subjects produce (S) -equol by dihydrodaidzein and tetrahydrodaidzein under the action of *Lactobacillus*. Subsequently, all these isoflavone aglycones were further converted and absorbed in the colon through reactions such as C-ring cleavage and dehydroxylation (Heinonen et al., 2004). In our study, the fourteen metabolites were able to enter the BL by monolayer, suggesting that these metabolites increase the uptake of substances and promote the absorption in the colon after the reactions of hydroxylation, methylation, glycine binding, sulfation and glucose binding. Studies have reported that proanthocyanidins are polymers of high molecular weight, and therefore oligomers larger than trimers are unlikely to be absorbed in the small intestine in their native form (Deprez et al., 2001). Meanwhile, genistin, as polymers of high molecular weight, was found on the AP. Apparently, it did not enter the BL through the monolayer, which clearly indicated that genistin could not be absorbed from the intestine. This was consistent with previous reports in the literature (Walle et al., 1999).

Some limitations in this study should be considered. The absorption experiment based on the Caco-2 cell monolayer showed that some metabolites were not added to the AP of the monolayer, which led to the inability to identify the absorption results of all metabolites. As one of the isoflavone, genistin presents good biological activities, such as anti-inflammatory and antioxidant activity. However, the biological activity of other fermentation metabolites is vague except for genistein. These problems and challenges will be revealed in further research.

## 5 Conclusion

In this study, we found that genistin can be converted into small-molecule metabolites by fecal fermentation. The results of 16S rRNA showed that dominant genera such as *Lactobacillus*, *Bifidobacterium,* and *Prevotella* played an irreplaceable role in the biotransformation reactions. Furthermore, we clarified the metabolic process and the trend of genistin content change in fecal bacteria through qualitative and quantitative studies. This has addressed limitations of previous studies that have only focused on qualitative identification and lacked quantitative research. Meanwhile, the results of *in vitro* culture of five dominant probiotics in feces and fermentation with genistin verified that bacteria in the feces could convert macromolecular components of traditional Chinese medicine into small molecule metabolites. Finally, based on the Caco-2 cell monolayer, small molecule metabolites from the fecal bacteria fermentation can be easily systematically absorbed by the mesentery and enter the internal circulation. These studies provide a reference value for explaining the transformation and absorption of flavonoid glycosides in the intestine.

## Data Availability

The datasets presented in this study can be found in online repositories. The names of the repository/repositories and accession number(s) can be found in the article/Supplementary Material.
