# Supplementary material for: Study on biotransformation and absorption of genistin based on fecal microbiota and Caco-2 cell
Source: Front Pharmacol. 2024 Oct 9;15:1437020. doi: 10.3389/fphar.2024.1437020 (PMC11496136; doi:10.3389/fphar.2024.1437020)
Supplement: Supplementary file 1 [file DataSheet1.docx]

**
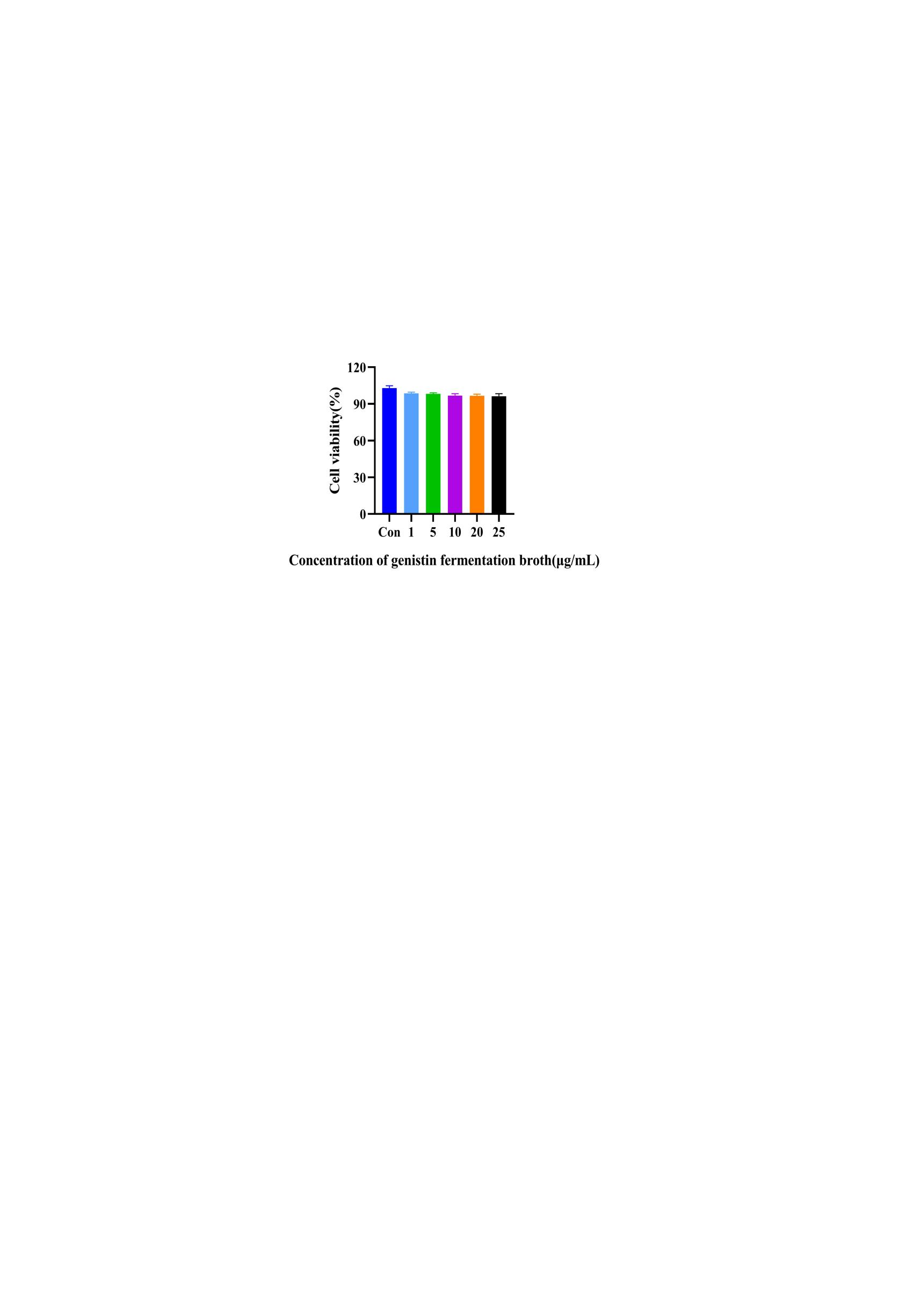
**

**FIGURE S1.** Determination of cell survival rate of genistin fermentation broth.

**
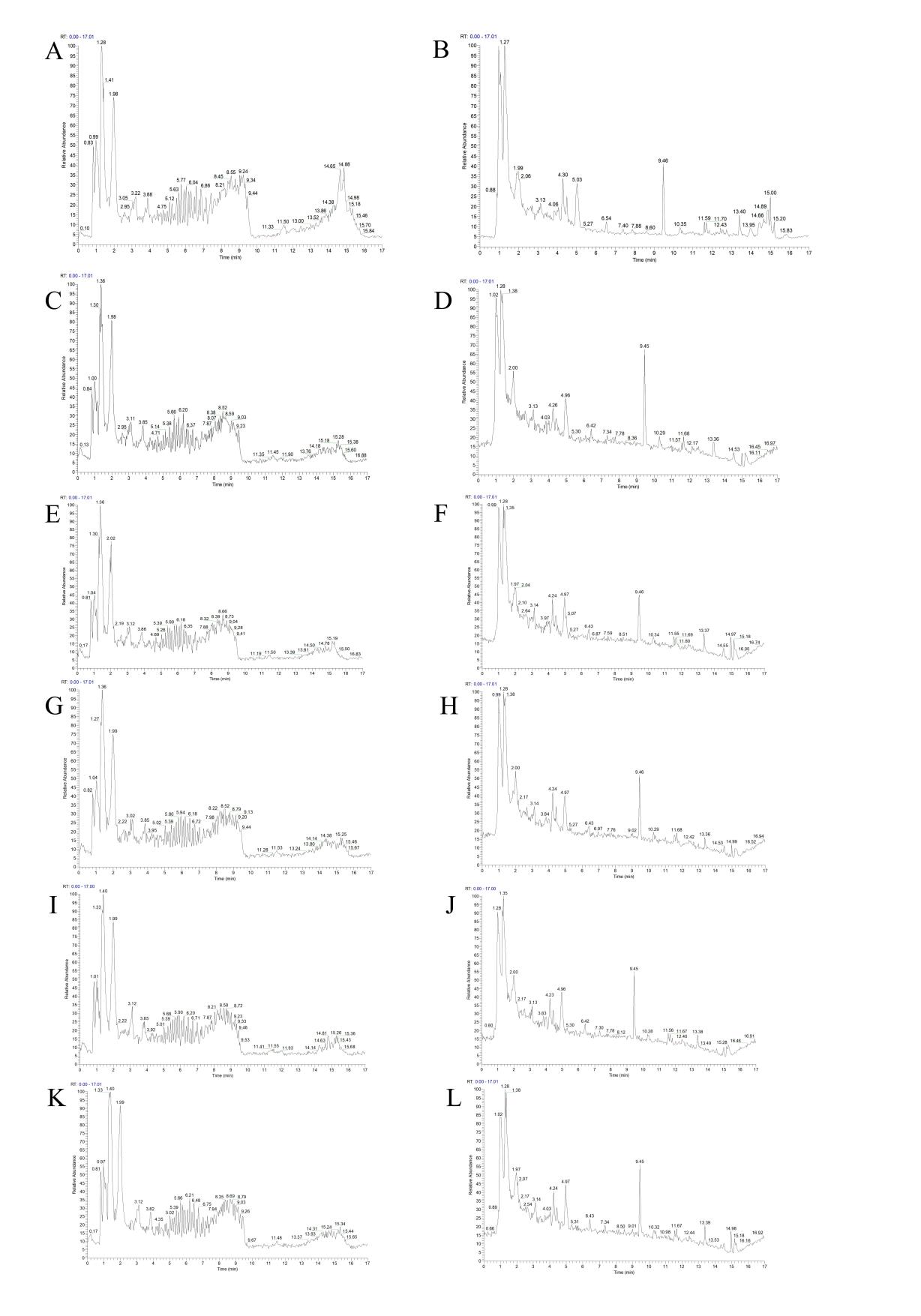
**

**FIGURE S2.** **(A,B)** Total ion current diagram of genistin fecal fermentation in positive and negative ion mode. The total ion flow diagram of genistin and five probiotics fermentation in positive and negative ion mode.**(C,D)** *Bifidobacterium longum*. **(E,F)** *Bifidobacterium adolescent*. **(G,H)** *Lactobacillus*. **(I,J)** *Lactobacillus plantarum B2*. **(K,L)** *Lactobacillus plantarum F1*.

**TABLE S1. Methodological Studies on Genistin**

**TABLE S1.1. Linear Function of Genistin and Genistein**

| Standards | Retention time/min | Regression equation^*^ | R² |
| --- | --- | --- | --- |
| Genistin | 4.96 | y = 675.48x + 20.774 | 0.9998 |
| Genistein | 16.067 | y = 1041.9x + 7.1306 | 0.9990 |

**Note:** ^*^x, Lg value of the standard solution concentration (lg mg/mL); y, Lg value of the peak area.

**TABLE S1.2. Precision, Reproducibility and Stability Studies of Genistin (mg/mL)**

|  | 1 | 2 | 3 | 4 | 5 | 6 | RSD |
| --- | --- | --- | --- | --- | --- | --- | --- |
| Precision investigation | 0.055316 | 0.053947 | 0.054435 | 0.054401 | 0.053939 | 0.054381 | 0.922% |
| Reproducibility investigation | 0.112820 | 0.111947 | 0.111524 | 0.113085 | 0.111553 | 0.113626 | 0.778% |
| Stability of sample | 0.080722 | 0.080555 | 0.080768 | 0.079775 | 0.080447 | 0.082851 | 1.188% |
| Stability of standards | 0.057669 | 0.057874 | 0.057792 | 0.058430 | 0.058283 | 0.059501 | 1.038% |

**TABLE S2. Distribution of Metabolites from Genistin Faecal Fermentation in *Lactobacillus* at Different Time Points**

| **Peaks** | **0 d** | **1 d** | **2 d** | **3 d** | **4 d** | **6 d** | **8 d** | **10 d** |
| --- | --- | --- | --- | --- | --- | --- | --- | --- |
| M0 | √ | √ | √ | √ | √ | √ | √ | √ |
| M1 | √ | √ | √ | √ | √ | √ | √ | √ |
| M2 | √ | √ | √ | √ | √ | √ | √ | √ |
| M3 | √ | √ | √ | × | × | × | × | × |
| M4 | √ | √ | √ | √ | √ | × | × | √ |
| M5 | × | × | × | × | × | × | × | × |
| M6 | × | × | × | × | √ | √ | × | × |
| M7 | √ | √ | √ | √ | √ | √ | √ | √ |
| M8 | × | × | × | × | × | × | × | × |
| M9 | × | × | × | × | × | × | × | × |
| M10 | √ | √ | √ | √ | √ | √ | √ | √ |
| M11 | × | × | × | × | × | × | × | × |
| M12 | √ | × | √ | × | √ | √ | √ | × |
| M13 | √ | √ | √ | √ | √ | × | × | √ |
| M14 | × | × | × | × | × | × | × | × |
| M15 | × | × | × | × | × | × | × | × |
| M16 | × | × | × | × | × | × | × | × |
| M17 | × | × | × | × | × | × | × | × |
| M18 | × | × | × | × | × | × | × | × |
| M19 | √ | × | √ | √ | √ | √ | √ | √ |
| M20 | × | × | × | × | × | × | × | × |
| M21 | √ | × | √ | × | √ | √ | √ | × |
| M22 | √ | √ | √ | √ | √ | √ | × | √ |
| M23 | × | √ | √ | √ | √ | √ | √ | √ |
| M24 | × | × | × | × | × | × | × | × |
| M25 | × | × | × | × | × | √ | × | × |
| M26 | × | × | × | × | × | × | × | × |
| M27 | × | × | × | × | √ | × | √ | × |
| M28 | × | × | × | × | × | × | × | × |
| M29 | × | × | × | × | × | × | × | × |
| M30 | × | √ | √ | √ | √ | √ | √ | √ |
| M31 | × | × | × | × | × | × | × | × |
| M32 | × | √ | √ | √ | √ | √ | √ | √ |
| M33 | × | √ | √ | √ | √ | √ | √ | √ |
| M34 | × | × | × | × | × | × | × | √ |
| M35 | × | × | × | × | × | × | × | × |
| M36 | √ | √ | × | × | √ | × | √ | √ |
| M37 | √ | √ | √ | √ | √ | √ | × | × |
| M38 | √ | √ | × | √ | √ | √ | √ | √ |
| M39 | √ | √ | × | √ | √ | √ | × | √ |
| M40 | × | × | × | × | √ | × | √ | × |
| M41 | √ | √ | √ | √ | √ | √ | √ | √ |
| M42 | √ | × | √ | × | √ | √ | √ | × |
| M43 | × | × | × | × | × | × | × | × |
| M44 | × | √ | √ | √ | √ | √ | √ | √ |
| M45 | × | √ | √ | × | √ | √ | √ | √ |

√: Metabolites were present. ×: Metabolites were not present.

**TABLE S3. Distribution of Metabolites from Genistin Faecal Fermentation in *Bifidobacterium Adolescent* at Different Time Points**

| **Peaks** | **0 d** | **1 d** | **2 d** | **3 d** | **4 d** | **6 d** | **8 d** | **10 d** |
| --- | --- | --- | --- | --- | --- | --- | --- | --- |
| M0 | √ | √ | √ | √ | √ | √ | √ | √ |
| M1 | √ | √ | √ | √ | √ | √ | √ | √ |
| M2 | √ | √ | √ | √ | √ | √ | √ | √ |
| M3 | × | × | × | × | × | × | × | × |
| M4 | √ | √ | √ | √ | √ | × | × | × |
| M5 | × | × | × | × | × | × | × | × |
| M6 | × | × | × | × | × | × | × | × |
| M7 | √ | √ | √ | √ | √ | √ | √ | √ |
| M8 | × | × | × | × | × | × | × | × |
| M9 | × | × | × | × | × | × | × | × |
| M10 | √ | √ | √ | √ | √ | √ | √ | √ |
| M11 | × | × | × | × | × | × | × | × |
| M12 | √ | × | × | √ | × | √ | √ | √ |
| M13 | √ | √ | √ | √ | √ | × | × | × |
| M14 | √ | × | × | × | × | × | × | × |
| M15 | √ | × | × | × | × | × | × | × |
| M16 | √ | × | × | × | × | × | × | × |
| M17 | × | × | × | × | × | × | × | × |
| M18 | √ | × | × | × | × | √ | × | × |
| M19 | √ | √ | √ | √ | √ | √ | √ | √ |
| M20 | √ | √ | × | × | × | × | × | × |
| M21 | √ | × | × | × | × | √ | √ | √ |
| M22 | √ | √ | √ | √ | √ | × | × | × |
| M23 | √ | √ | √ | √ | √ | √ | √ | √ |
| M24 | √ | × | × | × | × | × | × | × |
| M25 | √ | √ | × | × | × | √ | × | × |
| M26 | √ | × | × | × | × | × | × | × |
| M27 | × | × | × | × | × | × | × | × |
| M28 | × | × | × | × | × | × | × | × |
| M29 | × | × | × | × | × | × | × | × |
| M30 | × | √ | √ | √ | √ | √ | √ | √ |
| M31 | × | × | × | × | × | × | × | × |
| M32 | × | √ | √ | √ | √ | √ | × | × |
| M33 | √ | √ | √ | √ | √ | √ | √ | √ |
| M34 | √ | × | × | × | × | × | × | × |
| M35 | × | × | × | × | × | × | × | × |
| M36 | √ | √ | √ | √ | √ | √ | √ | √ |
| M37 | √ | √ | √ | √ | √ | × | × | × |
| M38 | √ | √ | × | √ | × | × | √ | √ |
| M39 | √ | √ | × | × | × | √ | √ | √ |
| M40 | × | × | × | × | × | × | × | × |
| M41 | √ | √ | √ | √ | √ | √ | √ | √ |
| M42 | × | × | × | √ | × | √ | √ | √ |
| M43 | × | √ | √ | √ | √ | √ | √ | √ |
| M44 | × | √ | √ | √ | √ | √ | × | × |
| M45 | × | × | × | × | √ | √ | √ | √ |

√: Metabolites were present. ×: Metabolites were not present.

**TABLE S4. Distribution of Metabolites from Genistin Faecal Fermentation in *Bifidobacterium Longum* at Different Time Points**

| **Peaks** | **0 d** | **1 d** | **2 d** | **3 d** | **4 d** | **6 d** | **8 d** | **10 d** |
| --- | --- | --- | --- | --- | --- | --- | --- | --- |
| M0 | √ | √ | √ | √ | √ | √ | √ | √ |
| M1 | √ | √ | √ | √ | √ | √ | √ | √ |
| M2 | √ | √ | √ | √ | √ | √ | √ | √ |
| M3 | × | × | × | × | × | × | × | × |
| M4 | √ | √ | √ | √ | √ | √ | √ | √ |
| M5 | × | × | × | × | × | × | × | × |
| M6 | √ | √ | √ | √ | √ | √ | √ | √ |
| M7 | √ | √ | √ | √ | √ | √ | √ | √ |
| M8 | × | × | × | × | × | × | × | × |
| M9 | × | √ | × | × | × | × | × | × |
| M10 | √ | √ | √ | √ | √ | √ | √ | √ |
| M11 | × | × | × | × | × | × | × | × |
| M12 | × | × | × | × | √ | × | × | √ |
| M13 | √ | √ | √ | √ | √ | √ | √ | √ |
| M14 | × | × | × | × | × | × | × | × |
| M15 | √ | × | × | × | × | × | × | × |
| M16 | × | × | × | × | × | × | × | × |
| M17 | × | × | × | × | × | × | × | × |
| M18 | × | √ | × | √ | × | × | × | × |
| M19 | √ | √ | √ | √ | √ | √ | √ | √ |
| M20 | √ | √ | × | √ | × | × | × | × |
| M21 | × | × | × | × | √ | × | × | √ |
| M22 | √ | √ | √ | √ | √ | √ | √ | √ |
| M23 | × | √ | √ | √ | √ | √ | √ | √ |
| M24 | √ | × | × | × | × | × | × | × |
| M25 | √ | × | √ | × | × | √ | × | √ |
| M26 | √ | × | × | × | × | × | × | × |
| M27 | × | × | √ | × | √ | √ | √ | √ |
| M28 | × | × | √ | √ | √ | √ | × | × |
| M29 | × | × | × | × | × | × | × | × |
| M30 | × | √ | √ | √ | √ | √ | √ | √ |
| M31 | × | × | × | √ | × | √ | √ | √ |
| M32 | × | √ | √ | √ | √ | √ | √ | √ |
| M33 | √ | √ | √ | √ | √ | √ | √ | √ |
| M34 | √ | × | × | × | × | × | √ | × |
| M35 | × | × | × | × | × | × | × | × |
| M36 | √ | × | × | × | √ | √ | √ | √ |
| M37 | √ | √ | √ | √ | √ | √ | √ | √ |
| M38 | √ | √ | √ | √ | √ | √ | √ | √ |
| M39 | √ | × | × | × | × | × | × | × |
| M40 | × | × | √ | × | √ | √ | √ | √ |
| M41 | √ | √ | √ | √ | √ | √ | √ | √ |
| M42 | × | × | × | × | √ | × | × | √ |
| M43 | × | √ | √ | √ | √ | √ | √ | √ |
| M44 | × | √ | √ | √ | √ | √ | √ | √ |
| M45 | × | √ | × | √ | √ | √ | √ | √ |

√: Metabolites were present. ×: Metabolites were not present.

**TABLE S5. Distribution of Metabolites from Genistin Faecal Fermentation in *Lactobacillus Plantarum F1* at Different Time Points**

| **Peaks** | **0 d** | **1 d** | **2 d** | **3 d** | **4 d** | **6 d** | **8 d** | **10 d** |
| --- | --- | --- | --- | --- | --- | --- | --- | --- |
| M0 | √ | √ | √ | √ | √ | √ | √ | √ |
| M1 | √ | √ | √ | √ | √ | √ | √ | √ |
| M2 | √ | √ | √ | √ | √ | √ | √ | √ |
| M3 | × | × | × | × | × | × | × | × |
| M4 | √ | √ | √ | √ | √ | √ | √ | √ |
| M5 | × | × | × | × | × | × | × | √ |
| M6 | × | × | × | × | × | × | × | × |
| M7 | √ | √ | √ | √ | √ | √ | √ | √ |
| M8 | × | × | × | × | × | × | × | × |
| M9 | × | √ | × | × | × | × | × | × |
| M10 | √ | √ | √ | √ | √ | √ | √ | √ |
| M11 | × | × | × | × | × | × | × | × |
| M12 | √ | × | × | √ | √ | √ | √ | √ |
| M13 | √ | √ | √ | √ | √ | √ | × | × |
| M14 | × | √ | × | × | √ | √ | √ | √ |
| M15 | × | × | × | × | × | × | × | × |
| M16 | × | × | × | × | × | × | × | × |
| M17 | × | × | × | × | × | × | × | × |
| M18 | √ | √ | √ | √ | √ | × | × | × |
| M19 | √ | √ | √ | √ | √ | √ | √ | √ |
| M20 | √ | × | × | × | × | × | × | × |
| M21 | √ | × | × | √ | √ | √ | √ | √ |
| M22 | √ | √ | √ | √ | √ | √ | √ | √ |
| M23 | × | √ | √ | √ | √ | √ | √ | √ |
| M24 | √ | × | × | × | × | × | × | × |
| M25 | × | × | × | × | × | × | × | × |
| M26 | √ | × | × | × | × | × | × | × |
| M27 | × | √ | × | × | × | × | √ | √ |
| M28 | × | × | × | √ | √ | √ | √ | √ |
| M29 | × | × | × | × | × | × | × | × |
| M30 | × | √ | √ | √ | √ | √ | √ | √ |
| M31 | × | √ | √ | × | × | × | √ | √ |
| M32 | × | √ | √ | √ | √ | √ | √ | √ |
| M33 | √ | √ | √ | √ | √ | √ | √ | √ |
| M34 | × | × | × | × | × | × | × | × |
| M35 | × | × | × | × | × | × | × | × |
| M36 | √ | × | × | × | √ | √ | √ | √ |
| M37 | √ | √ | √ | √ | √ | √ | √ | √ |
| M38 | √ | √ | √ | √ | √ | √ | √ | √ |
| M39 | √ | × | × | × | × | √ | √ | √ |
| M40 | × | √ | × | × | × | × | √ | √ |
| M41 | √ | √ | √ | √ | √ | √ | √ | √ |
| M42 | √ | × | × | √ | √ | √ | √ | √ |
| M43 | × | √ | √ | √ | √ | √ | √ | √ |
| M44 | × | √ | √ | √ | √ | √ | √ | √ |
| M45 | × | × | √ | √ | √ | √ | √ | √ |

√: Metabolites were present. ×: Metabolites were not present.

**TABLE S6. Distribution of Metabolites from Genistin Faecal Fermentation in *Lactobacillus Plantarum B2* at Different Time Points**

| **Peaks** | **0 d** | **1 d** | **2 d** | **3 d** | **4 d** | **6 d** | **8 d** | **10 d** |
| --- | --- | --- | --- | --- | --- | --- | --- | --- |
| M0 | √ | √ | √ | √ | √ | √ | √ | √ |
| M1 | √ | √ | √ | √ | √ | √ | √ | √ |
| M2 | √ | √ | √ | √ | √ | √ | √ | √ |
| M3 | × | × | × | × | × | × | × | × |
| M4 | √ | √ | √ | √ | √ | √ | √ | √ |
| M5 | × | × | × | × | × | × | × | × |
| M6 | × | × | × | × | × | × | × | × |
| M7 | √ | √ | √ | √ | √ | √ | √ | √ |
| M8 | × | × | × | × | × | × | × | × |
| M9 | × | × | × | × | × | × | × | × |
| M10 | √ | √ | √ | √ | √ | √ | √ | √ |
| M11 | × | × | × | × | × | × | × | × |
| M12 | × | × | × | × | × | × | × | × |
| M13 | × | √ | √ | √ | × | √ | √ | × |
| M14 | × | × | × | × | × | × | × | × |
| M15 | × | × | × | × | × | × | × | × |
| M16 | × | × | × | × | × | × | × | × |
| M17 | × | × | × | × | × | × | × | × |
| M18 | × | × | × | × | × | × | × | × |
| M19 | √ | √ | √ | √ | √ | √ | √ | √ |
| M20 | × | × | × | × | × | × | × | × |
| M21 | × | × | × | × | × | × | × | × |
| M22 | × | √ | √ | √ | √ | √ | √ | × |
| M23 | × | √ | √ | √ | √ | √ | √ | √ |
| M24 | × | × | × | × | × | × | × | × |
| M25 | × | × | × | × | × | × | × | × |
| M26 | √ | √ | √ | √ | √ | × | × | × |
| M27 | × | × | × | √ | √ | √ | √ | √ |
| M28 | × | × | × | × | × | × | × | × |
| M29 | × | × | × | × | × | × | × | × |
| M30 | × | √ | √ | √ | √ | √ | √ | √ |
| M31 | × | × | × | × | × | × | × | × |
| M32 | × | √ | √ | √ | √ | √ | √ | √ |
| M33 | √ | √ | √ | √ | √ | √ | √ | √ |
| M34 | × | × | × | × | × | × | × | × |
| M35 | × | × | × | × | × | × | × | × |
| M36 | √ | √ | × | × | √ | × | √ | √ |
| M37 | × | × | × | × | × | × | × | × |
| M38 | × | × | × | × | × | × | × | × |
| M39 | √ | × | × | × | √ | × | × | × |
| M40 | × | × | × | √ | √ | √ | √ | √ |
| M41 | √ | √ | √ | √ | √ | √ | √ | √ |
| M42 | × | × | × | × | × | × | × | × |
| M43 | × | √ | √ | √ | √ | √ | √ | √ |
| M44 | × | √ | √ | √ | √ | √ | √ | √ |
| M45 | × | √ | √ | × | √ | √ | √ | √ |

√: Metabolites were present. ×: Metabolites were not present.
